# Assessing the association between age at first sexual intercourse and migraine: a Mendelian randomization study

**DOI:** 10.3389/fneur.2024.1347831

**Published:** 2024-02-06

**Authors:** Guoliang Zhu, Miao Wang, Yawen Wang, Fanyi Kong

**Affiliations:** ^1^Department of Neurology, The Affiliated Hospital of Yunnan University, Kunming, Yunnan, China; ^2^School of Clinical Medicine, Dali University, Dali, Yunnan, China; ^3^Department of Neurology, West China Hospital, Sichuan University, Chengdu, China; ^4^Department of Radiology, The Affiliated Hospital of Yunnan University, Kunming, Yunnan, China

**Keywords:** migraine, Mendelian randomization, causal association, age at first sexual intercourse, GWAS

## Abstract

**Background and objectives:**

As indicated by observational and genetic variation studies, age at first sexual intercourse (AFS) may be associated with migraine attack, but there is a lack of evidence from real-world studies due to ethical concerns. Therefore, we conducted a Mendelian randomization study to determine the causal relationship between AFS and migraine.

**Methods:**

We extracted instrumental variables from summary data of a genome-wide association study (GWAS) on AFS and migraine and then conducted two-sample Mendelian randomization analyses. GWAS data for AFS and migraine were obtained from 397,338 unrelated individuals (214,547 females and 182,791 males) and 306,314 individuals (18,477 patients and 287,837 control individuals), respectively.

**Results:**

There was a causal relationship between AFS and risk for migraine (odds ratio (OR) = 0.73, 95% confidence interval (CI) [0.61 to 0.86]), both for migraine with aura (MWA; OR = 0.72, 95% CI [0.58 to 0.89]) and migraine without aura (MOA; OR = 0.66, 95% CI [0.51 to 0.86]). Stratified analyses provided suggestive evidence of the causal relationship between delayed AFS and a decreased risk of migraine for both males (OR = 0.71, 95% CI [0.59 to 0.84]) and females (OR = 0.73, 95% CI [0.61 to 0.89]). Reverse Mendelian randomization did not reveal any effect of migraine on AFS (*p* > 0.05). No pleiotropy was detected.

**Discussion:**

A delayed AFS is a protective factor against migraine (for both MWA and MOA) in both males and females. This causal relationship indicates the presence of extracranial regulatory pathways of migraine.

## Introduction

Migraine is the second most disabling disease globally, and 45.1 million migraine sufferers are estimated to be limited by this disease in their daily lives (1). The pathogenesis of migraine is currently unclear, but increasing evidence suggests that sex hormones trigger episodes of migraine, especially in female patients (2).

Headache associated with sexual activity (HAWSA), which refers to an abrupt explosive headache during orgasm only or a worsening of headache intensity with increasing sexual excitement, occurs in approximately 1% of the population (3). The HAWSA was recognized as a high comorbidity of migraine (25%) (4). It has been discovered that there is a bilateral association between HSA and migraine (5). A genetic variation study revealed that the later the age at first sexual intercourse (AFS) was, the lower the risk of cardiovascular disease (6). In real-world studies, cardiovascular disease has been shown to be associated with migraine (7). Additionally, a genetic association of migraine with type 2 diabetes and blood lipids was also identified (8), and it was claimed that the risk of type 2 diabetes and elevated low-density lipoprotein increases with decreasing AFS (9).

We hypothesize that there is an undiscovered association between AFS and migraine, which is not widely known among the general public and needs explicit clarification. However, it is challenging to conduct a randomized controlled trial or a real-world observational study because it is morally and legally unacceptable to have underage individuals engage in sexual activities.

Mendelian randomization (MR) analysis uses instrumental variables (IVs), typically single nucleotide polymorphisms (SNPs), to explore causal effects between exposure and outcome. MR analysis can significantly reduce the impact of uncontrolled confounders in observational studies, making it suitable for use in situations where it is not feasible to implement randomized controlled trials. Based on the promising efficacy of MR for exploring causal relationships and the aforementioned reasons under comprehensive consideration, we aimed to explore the potential causal relationship between AFS and migraine by using MR analysis.

## Methods

We followed the STROBE-MR Statement (10) in reporting the present study. Ethics approval and informed consent were not needed for this study, as the data used in this study were obtained from the original study, which was approved by their ethics committee (11).

### Study design

Exposure was defined as AFS, and the outcome was defined as migraine. We planned to use SNPs strongly associated with exposure as IVs. Of note, IVs needed to meet the following criteria: (1) IVs were strongly associated with the exposure; (2) IVs were unrelated to any confounding factors to outcome; and (3) IVs affected the outcome only through the exposure pathway (10).

### Data sources

We obtained genetic data from a genome-wide association study (GWAS) of 397,338 unrelated individuals in the GWAS Catalog.^1^ Individuals under 12 years old were excluded. The methodological quality of the primary literature that provided the GWAS data (11) was assessed, and it was determined to be of high quality; the GWAS data were used by the authors of a previously published manuscript (6).

The migraine GWAS data were obtained from the ninth edition of the Finngen Biobank as of May 2023.^2^ We selected the migraine cohort, which included all patients with migraine coded as G6-MIGRAINE. The diagnosis of migraine was made according to the International Classification of Diseases (ICD)-8, ICD-9, and ICD-10 codes. Stratification analysis was performed by extracting GWAS data for the migraine with aura (MWA) and migraine without aura (MOA) subgroups in the migraine cohort. The outcome data for the present MR analysis were GWAS data for MWA (7,917 cases and 287,837 controls) and MOA (6,730 cases and 287,837 controls). Table 1 shows the datasets that we included.

**Table 1:** Characteristics of GWAS datasets.

Trait	Study	Data source	Sample size	Ancestry	Definition
**Exposure**					
AFS	Mills MC et al.	GWAS catalog	397,338 individuals	European	Not applicable
AFSM	Mills MC et al.	GWAS catalog	182,791 individuals	European	Not applicable
AFSF	Mills MC et al.	GWAS catalog	214,547 individuals	European	Not applicable
**Outcome**					
Migraine	FinnGen (Release 9)	FinnGen study	18,477 cases and 287,837 controls	European	ICD-8, ICD-9 and ICD-10
MWA	FinnGen (Release 9)	FinnGen study	7,917 cases and 287,837 controls	European	ICD-8, ICD-9 and ICD-10
MOA	FinnGen (Release 9)	FinnGen study	6,730 cases and 287,837 controls	European	ICD-8, ICD-9 and ICD-10

GWAS, genome-wide association study; AFS, age at first sexual intercourse; AFSM, age at first sexual intercourse of male; AFSF, age at first sexual intercourse of female; MWA, migraine with aura; MOA, migraine without aura; ICD, International Classification of Diseases.

The present study is a *post hoc* analysis using secondary analyses of published data. As only summary statistics rather than individual-level data were obtained from the GWAS data, comprehensive statistical information regarding sample and genetic variant missingness was not possible.

In this study, exposures and outcomes were derived from two different large-scale GWAS databases, with a low probability of sample overlap. In addition, a study showed that single-sample MR study results with a large sample size (*n* = 300,000) were similar to those of double-sample studies, and the sample size we included met this criterion (12).

To ensure the possibility of a strong association between IVs and exposures, we excluded SNPs with a *p*-value ≥ 5 × 10^−8^ related to AFS (13). We selected SNPs suitable for serving as IVs in the following steps: (1) we extracted SNPs strongly associated with exposure from GWAS by using the TwoSampleMR package (version 0.5.6) in R software (version 4.3); (2) to avoid the impact of linkage disequilibrium on the results, we set clump as *r*^2^ = 0.001 and kb = 10,000; (3) we excluded palindromic sequences in SNPs; (4) SNPs that were strongly associated with exposure were matched with outcome GWAS; (5) to exclude the effect of confounding factors, we searched for SNPs that met the above criteria in PhenoScanner^3^ and excluded SNPs that may be affected by confounding factors (MR hypothesis II) (14); and (6) we used the F-statistic to evaluate weak instrument variable effects. The calculation formula was as follows: F = $\frac{\beta^{2}}{SE^{2}}$ (15) ( β = effect size of exposure; SE = standard error of β ). When the F-statistic was less than 10, we considered that there was a weak instrumental variable and that IV needed to be eliminated, or MR analysis was not recommended (16). We calculated the F-statistic for each IV and found no occurrence of *F* < 10, with a minimum of 27.44 and a maximum of 171.49.

### MR analysis

We used the Wald ratio method to obtain causal effect estimates of AFS on migraine risk. When a small number of SNPs were not detected in the outcome, these SNPs were excluded (15). We used three testing methods to conduct the present experiment. The random-effects Inverse-variance weighted (IVW) approach was considered the primary analysis method (17). We also used the weighted median and MR–Egger methods as supplementary analysis methods (18, 19).

**Figure 1:**
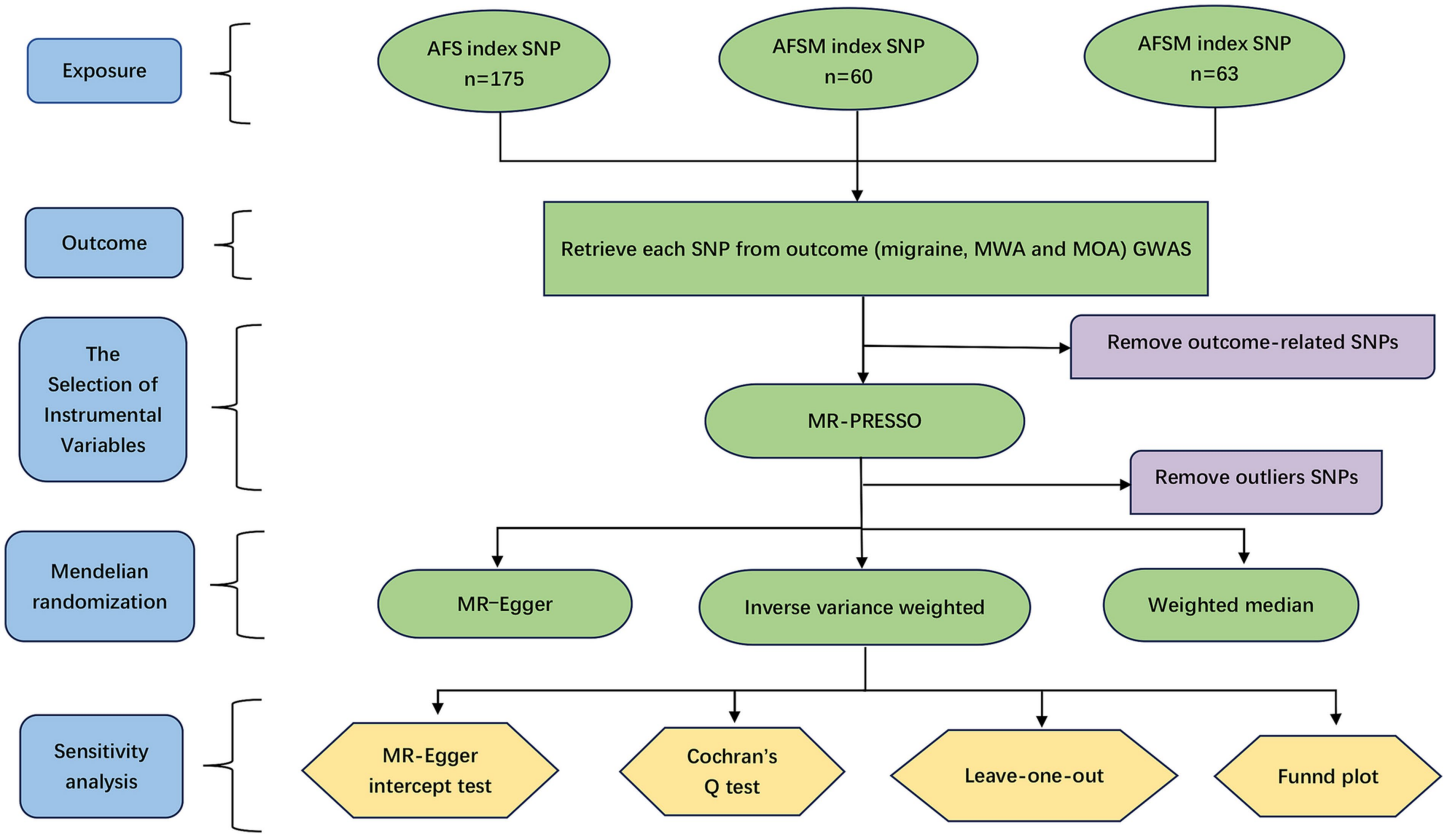
The research framework. AFS, age at first sexual intercourse; AFSM, age at first sexual intercourse of male; AFSF, age at first sexual intercourse of female; MWA, migraine with aura; MOA, migraine without aura; SNP, single nucleotide polymorphism.

We additionally conducted a robust analysis to avoid the influence of outliers (robust IVW and robust MR–Egger). The MR–Egger intercept test was used to assess and determine the existence of horizontal pleiotropy. The Mendelian randomization pleiotropy residual sum and outlier (MR-PRESSO) can sum each SNP’s residuals to evaluate the magnitude of horizontal pleiotropy. After adjusting for horizontal pleiotropy, the IVW primary analysis method results were obtained. The overall horizontal pleiotropy of the IVs was evaluated by MR-PRESSO global assessment, while the abnormal SNPs that caused overall horizontal pleiotropy were considered by MR-PRESSO outlier assessment. In addition, the heterogeneity level was assessed by using Cochran’s *Q* statistic. We also conducted a leave-one-out analysis by calculating the remaining SNP effect after excluding each SNP one by one. The leave-one-out method was used to evaluate whether significant SNPs affected our results when needed. A funnel plot was drawn to assess the existence of directional pleiotropy. The framework of the present research is displayed in Figure 1.

To ensure the reliability of the study results, we evaluated the influence of potential confounders. In the original study providing AFS GWAS data, researchers calculated the genetic association of AFS with six related categories containing 25 characteristics according to sex. Age at first menarche, age at menopause, age at starting oral contraceptives, teenage pregnancy, severe depression, weekly alcohol consumption, age at starting smoking, marijuana use, and body mass index (BMI) may serve as mediators of AFS contributing to migraine. However, the results of the overall genetic association revealed that AFS is genetically associated with age at smoking initiation and teenage pregnancy (11). In addition, we also searched for the genetic association of each IV and excluded IVs that were genetically associated with confounding factors. By excluding the abovementioned confounders, the reliability and stability of our present results were verified. PhenoScanner, a platform with comprehensive information on the association of genotype and phenotype (available at http://www.phenoscanner.medschl.cam.ac.uk), was used to query for traits related to the IVs (*P* < 5 × 10^−8^), and those IVs were subsequently excluded as potential pathways outside the AFS that may affect migraine. In previous MR studies, insulin-like growth factor 1 and diastolic blood pressure were found to be causally related to migraine, so we excluded SNPs associated with those traits (20, 21). Available studies have also shown that migraine is related to body fat percentage (22). Therefore, SNPs associated with those traits were also excluded. Finally, reverse MR was performed to evaluate whether migraine would affect AFS. Since migraine is a binary variable, in forward MR analysis, the results are presented in the form of odds ratios (ORs), which quantify the change in migraine risk with an additional increase in the standard deviation (SD) of the AFS. In reverse MR analysis, the results are presented as β values, which represent the number of SDs of AFS change caused by migraine.

### Statistics

We calculated the power of the MR analysis using an online tool (23) (mRnd: Power calculations for Mendelian Randomization).^4^ The basis of a type I error rate was set at 0.05. The proportion of variance explained by the SNPs for exposure was 5.8% (11). The detectable OR based on the GWAS of migraine with 80% power was less than 0.912 (or greater than 1.089) according to the calculation. The detectable ORs of MWA and MOA based on the GWAS were less than 0.869 (or greater than 1.132) and less than 0.857 (or greater than 1.143), respectively. All hypothesis testing was 2-sided. Given that stratified analysis was used, nine MR analyses were performed in this study. The nominal significance *p*-value was set to 0.05, and after Bonferroni correction, the *p*-value was adjusted to 0.05/9 (0.0056).

All the statistical analyses were performed using STATA 17.0 software (Stata Statistical Software: College Station, TX: Stata Corp LP) and R (version 4.3). MR analysis was conducted by the TwoSampleMR (version 0.5.6), Mendelian Randomization (version 0.6.0) and MR-PRESSO (version 1.0) packages in R software. The execution date was May 2023.

## Results

The migraine genetic data on a total of 397,338 unrelated European individuals (54% females and 46% males) from the GWAS Catalog and 306,314 individuals (including 6% patients and 94% control individuals) from the Finngen Biobank were ultimately included in the present study. Of the 16,516,521 SNPs, a total of 175 SNPs (including 32 proxy SNPs) were identified as strongly associated with AFS. The age at first sexual intercourse of males (AFSM) and females (AFSF) were included in 60 (including 11 proxy SNPs) and 63 (including 8 proxy SNPs) SNPs, respectively. These SNPs were not strongly associated with the outcomes (*p* > 5 × 10^−8^). The SNP information is displayed in Supplementary Tables 1–3.

### Relationship between AFS and migraine

**Figure 2:**
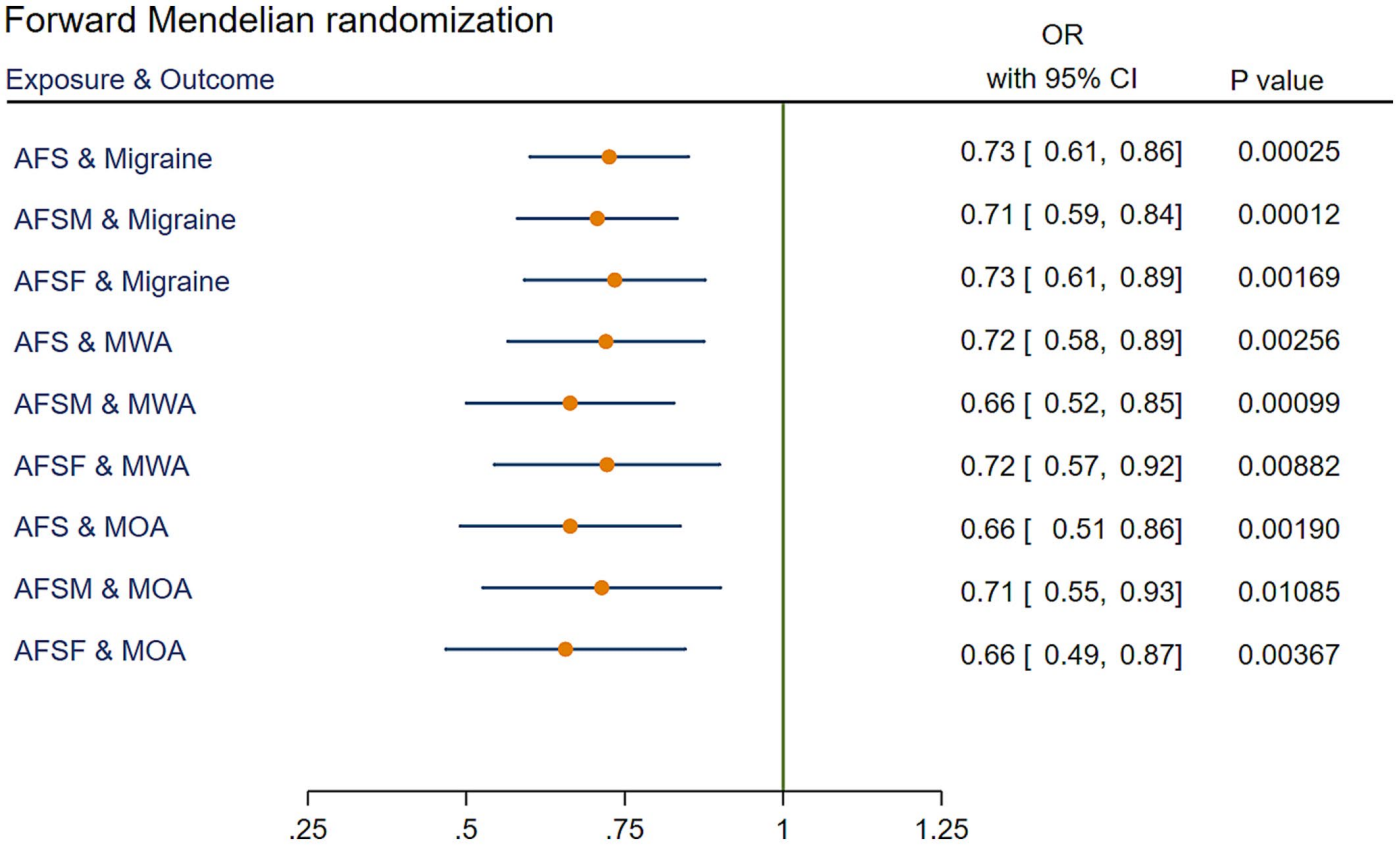
IVW results of forward Mendelian randomization. AFS, age at first sexual intercourse; AFSM, age at first sexual intercourse of male; AFSF, age at first sexual intercourse of female; MWA, migraine with aura; MOA, migraine without aura; MR, Mendelian randomization; IVW, Inverse variance weighted; OR, odds ratio; CI, confidence interval.

MR analysis showed that AFS had a significant causal relationship with migraine. The IVW method showed a significant result (OR = 0.73, 95% confidence interval [CI] [0.61 to 0.86], *p* = 2.49 × 10^−4^). Similar results were obtained using the weighted median method (OR = 0.76, 95% CI [0.61 to 0.95], *p =* 1.33 × 10^−2^). GWAS data with MWA and MOA outcomes were extracted from the Finngen Biobank for MR analysis. The results showed that AFS was significantly associated with both MWA (OR = 0.72, 95% CI [0.58 to 0.89], *p* = 2.56 × 10^−3^) and MOA (OR = 0.66, 95% CI [0.51 to 0.86], *p* = 1.9 × 10^−3^). The MR results are presented in Figure 2 and Supplementary Table 4.

### Relationship between AFSM and migraine

The IVW results revealed a significant association between AFSM and migraine (OR = 0.71, 95% CI [0.59 to 0.84], *p* = 1.22 × 10^−4^). These positive results also appeared in the MR analysis for MWA (OR = 0.71, 95% CI [0.59 to 0.84], *p* = 9.94 × 10^−4^) and WOA (OR = 0.71, 95% CI [0.55 to 0.93], *p* = 1.08 × 10^−2^). The MR results are presented in Figure 2 and Supplementary Table 4.

### Relationship between AFSF and migraine

Through MR analysis, we found a causal relationship between AFSF and migraine (the result of the IVW method showed OR = 0.73, 95% CI [0.61 to 0.89], *p* = 1.69 × 10^−3^). Stratified analysis determined that AFSF had a significant protective effect against both MWA (OR = 0.72, 95% CI [0.57 to 0.92], *p* = 8.82 × 10^−3^) and MOA (OR = 0.66, 95% CI [0.49 to 0.87], *p* = 3.67 × 10^−3^). The MR results are presented in Figure 2 and Supplementary Table 4.

**Figure 3:**
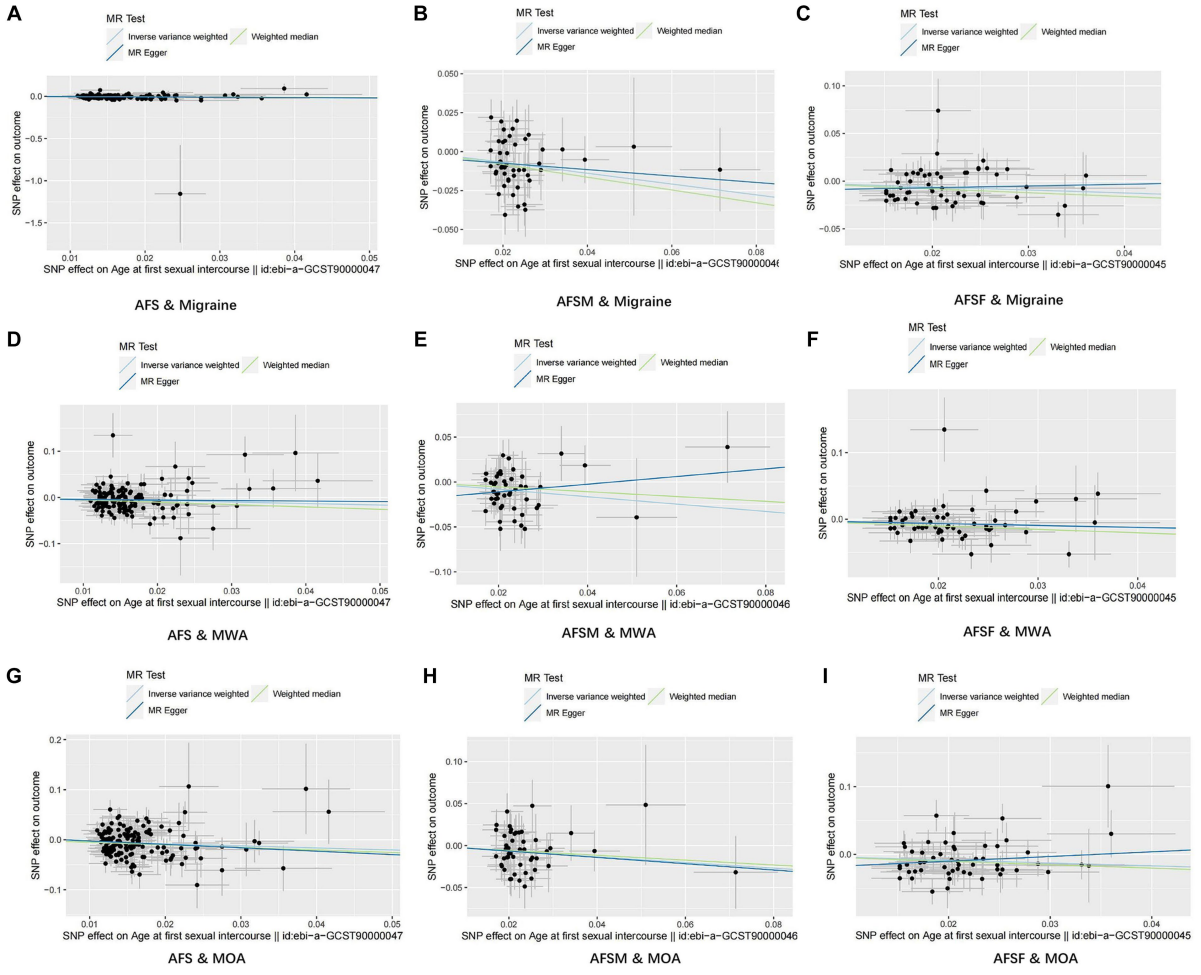
Results of scatter plots. **(A)** AFS & Migraine; **(B)** AFSM & Migraine; **(C)** AFSF & Migraine; **(D)** AFS & MWA; **(E)** AFSM & MWA; **(F)** AFSF & MWA; **(G)** AFS & MOA; **(H)** AFSM & MOA; **(I)** AFSF & MOA. AFS, age at first sexual intercourse; AFSM, age at first sexual intercourse of male; AFSF, age at first sexual intercourse of female; MWA, migraine with aura; MOA, migraine without aura.

For visualization of the results, we plotted scatter plots, which are displayed in Figure 3.

### Sensitivity analysis

The MR–Egger method did not detect a significant association in any results of MR analysis. No horizontal pleiotropism was found in the MR–Egger regression (*P* < 0.05). Furthermore, the MR-PRESSO method did not identify any outlier SNPs. The results are presented in Supplementary Figure 1.

By using Cochran’s *Q* statistic, we found heterogeneity in the results of AFS and migraine (*p* = 4.02 × 10–7), AFSF and migraine (*p* = 3.41 × 10–2), and AFS and MOA (*p* = 2.16 × 10–4). There was no apparent heterogeneity in the other reported results (*p* < 0.05). The data are shown in Supplementary Figure 1.

The leave-one-out analysis did not reveal that any SNPs significantly impacted the results. The results of the leave-one-out analysis are shown in Supplementary Figure 2. No significant bias was found in the funnel plot, as shown in Supplementary Figure 3.

**Figure 4:**
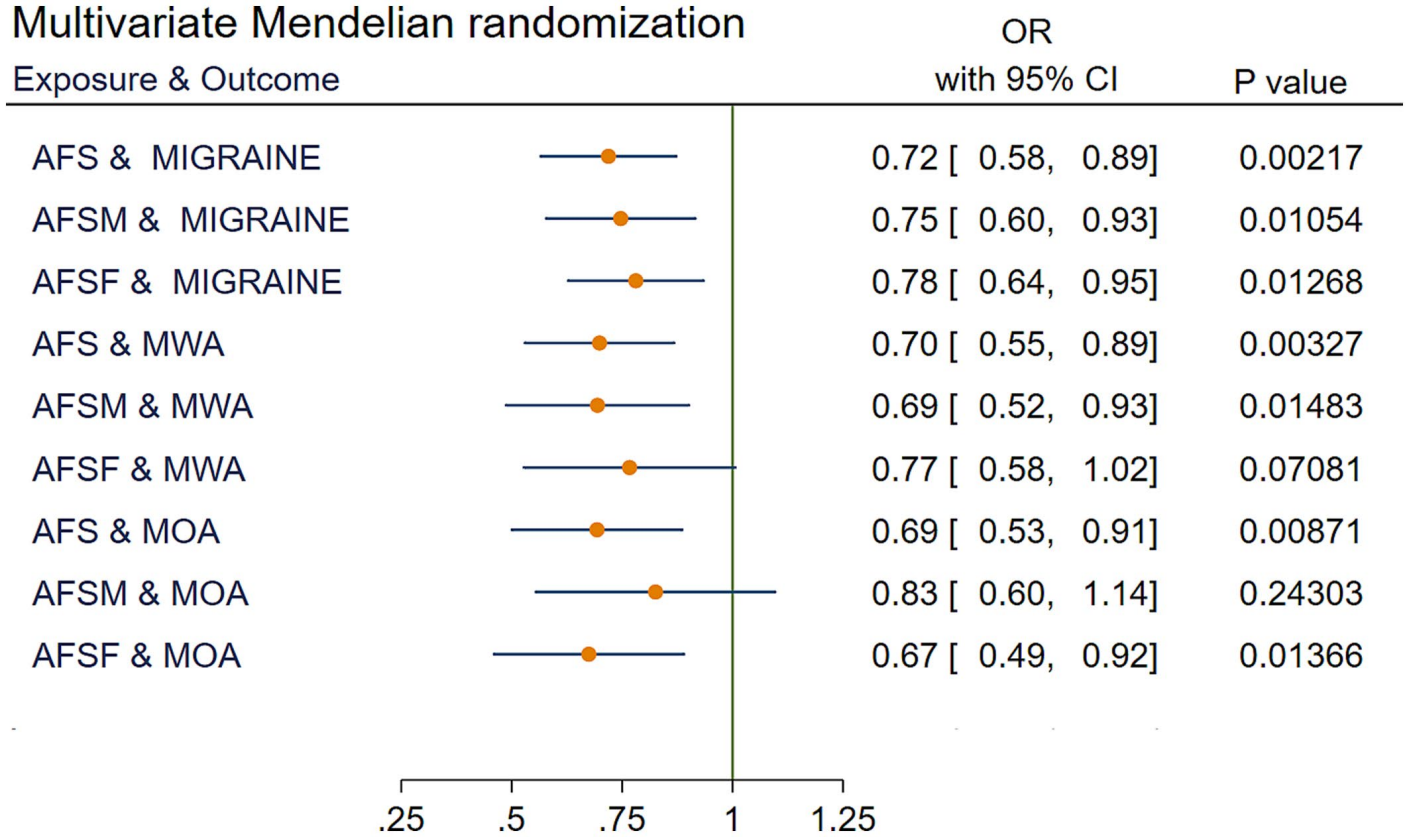
IVW results of multivariate Mendelian randomization. AFS, age at first sexual intercourse; AFSM, age at first sexual intercourse of male; AFSF, age at first sexual intercourse of female; MWA, migraine with aura; MOA, migraine without aura; MR, Mendelian randomization; IVW, Inverse variance weighted; OR, odds ratio; CI, confidence interval.

After excluding SNPs that may cause interference with confounding factors to migraine, the causal relationships in AFSM and MOA (OR = 0.83, 95% CI [0.60 to 1.14], *p* = 2.43 × 10^−1^) and AFSF and MWA (OR = 0.77, 95% CI [0.58 to 1.02], *p* = 7.08 × 10^−2^) were no longer statistically significant. The results are shown in Figure 4 and Supplementary Table 5.

**Figure 5:**
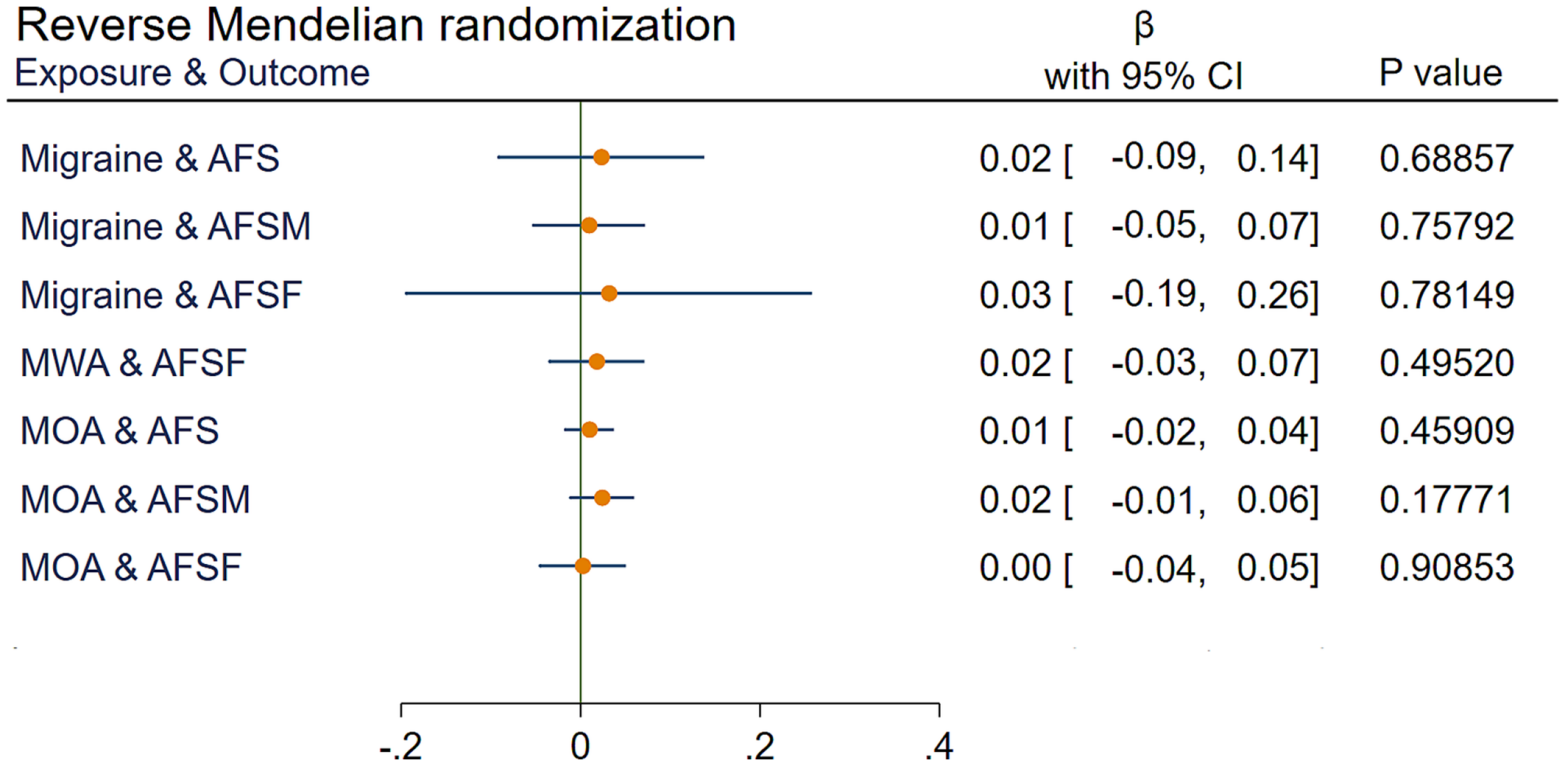
IVW results of reverse Mendelian randomization. AFS, age at first sexual intercourse; AFSM, age at first sexual intercourse of male; AFSF, age at first sexual intercourse of female; MWA, migraine with aura; MOA, migraine without aura; MR, Mendelian randomization; IVW, Inverse variance weighted; β , the estimated effect; CI, confidence interval.

Reverse MR analyses determined that migraine and the MOA had no significant impact on AFS, AFSF, or the AFSM (all *p* > 0.05). MWA had a nonsignificant effect on AFSF (*p* > 0.05). Due to the limitations of IVs, the impact of MWA on AFS and AFSF cannot be verified. The results are shown in Figure 5.

## Discussion

To our knowledge, this is the first MR study to explore the causal relationship between AFS and migraine. Through two-sample Mendelian randomization analyses using data from large-scale GWAS databases, we determined that an earlier age at first sexual intercourse is related to susceptibility to migraine attack. To address clinical interest, we manually classified the exposures into AFS, AFSF, and AFSM. Correspondingly, the outcomes were classified into migraine, MWA, and MOA when performing pairwise MR analyses between each exposure and outcome. All the *p* values of the results were less than 0.05, except for two subgroups (AFSF and MOA and AFSM and MWA) whose *p* values did not meet the Bonferroni-corrected standard (adjusted *p* < 0.0056; see Supplementary Figure 4). Hence, the above results should be interpreted with caution, as the possibility of false-positive results produced by AFSF and MOA and AFSM and MWA might exist. As indicated by reverse MR analyses, migraine has no causal relationship with AFS. Then, the potential reciprocal effect between migraine and ASF could be excluded.

By excluding SNPs that are associated with other related factors of migraine, MR analyses on exposures and outcomes suggested that two results (AFSM and MOA and AFSF and MWA) were no longer statistically significant. After adjustment for age at menarche (a marker of puberty and estrogen changes), age of starting smoking, age of starting oral contraceptives, teenage pregnancy, and BMI in our analyses, the results of the mediation analyses still showed an association between AFS and migraine. This finding suggested that AFS is not merely an accompanying phenomenon influenced by factors such as puberty, hormonal changes, unhealthy behaviors (smoking, drinking), contraception, and pregnancy. In other words, the causal relationship between AFS and migraine is robust.

We determined that later AFS has a protective effect against migraine. This may be attributed to sexual intercourse leading to increased dopamine secretion in the hypothalamus, which increases the levels of cyclic adenosine monophosphate and protein kinase A (24, 25). Both are thought to be responsible for migraine attacks (26). Testosterone produced during sexual intercourse was found to increase the level of cyclic guanosine monophosphate, which is thought to be another contributor to MOA attacks (27, 28). Finding from an animal study has shown that mice experience a decrease in the trigeminal pain threshold and an increase in photosensitivity during the estrus phase, indicating that sexual activity may trigger migraine attacks as early as during the estrus phase (29). In addition, penile-vaginal intercourse increases parasympathetic activity, which induces migraine by promoting the degranulation of mast cells (30, 31). Observational studies have shown a bilateral association between HAWSA and migraine (5). One study also reported patients who experienced migraine aura during sexual climax (32). All the above mentioned mechanisms theoretically supported the rationality of our present findings, and it is reasonable to believe that sexual activity has the potential to promote the occurrence of migraine.

In the stratification analysis, we found that a later AFS has a protective effect on MWA. Apart from the mechanism of migraine attack mentioned above, cortical spreading depression (CSD) inhibition is considered the main physiological mechanism of MWA, and susceptibility to CSD is modulated by hormones (ovarian and testicular) (33). Therefore, it is reasonable to infer that experiencing sexual activity at an early age involves migraine attack mechanisms, including migraine with aura. Delaying AFS is suggested for individuals with a high risk of migraine.

Another potential mechanism attributed to AFS and migraine may be related to the involvement of pituitary adenylate cyclase-activating polypeptide (PACAP), an essential target for migraine (34). In addition, PACAP is involved in the spermatogenesis process of mice (35), as well as in the production of steroids and the development of ovarian follicles (36). With premature sexual intercourse, the process of sperm production and the secretion of sex hormones are more frequently activated, hence promoting the increased synthesis of PACAP and thereby inducing migraine.

Our present finding on the causal relationship between AFS and migraine attacks could also be supported by real-world studies with respect to lifestyle changes. A cross-sectional study in Denmark revealed that AFS was associated with emergency contraceptive pill use (37). When hormonal contraceptive users are not taking hormonal contraceptives, migraine attacks worsen and last longer (38). Moreover, AFS will cause teenagers to bear more psychological pressure and feelings of vulnerability, which is considered a trigger factor for migraine (39, 40). Despite this indirect evidence, our present findings need future confirmation in a cohort study.

Sensitivity analysis was performed using conventional analysis methods and adjusted for the definition of association between exposure and SNPs. The MR-PRESSO method did not reveal evidence suggesting horizontal pleiotropy, indicating that the IVs did not affect migraine through pathways other than AFS. When Cochran’s *Q* test was used to explore heterogeneity across genetic variants in subgroups of AFS and migraine, AFSF and migraine, and AFS and MOA, we adopted a random-effects model to reduce the impact of heterogeneity on the results. By using leave-one-out analysis, we found that no single IV influenced the results, thus indicating the robustness of our results. Furthermore, there was an absence of directional pleiotropy, as exhibited in the funnel plot. Taken together, the sensitivity analyses verified that our present MR analysis results are reliable and stable.

Our research provides valuable recommendations for clinical work. First, AFS may participate in migraine attacks mediated through multiple pathways, including PACAP, dopamine and sex hormones. In addition, migraine sufferers, especially adolescents and those at high risk for migraine, are not encouraged to engage in sexual activity at an early age. Finally, well-designed cohort studies are encouraged in the future to explore the relationship between sexual activity and migraine. Taken together, these findings suggest that delaying the age at first sexual intercourse is recommended for adolescents. Patients with HAWSA might have a higher risk of migraine, but this topic needs further investigation in the future through prospective cohort studies.

There should be an awareness of the limitations of the present study. First, the GWAS data derived from European populations limit the generalization of our findings to people of other ethnic groups. Second, we were unable to assess the impact of unmeasured confounders on the results, which is encountered as a challenging issue in almost all MR analyses. Third, as information on the exact age at first sexual intercourse was not available from GWAS data, we could not provide a particular recommendation on age at which sexual activity commenced. Finally, the diagnosis of migraine or MWA based on the ICD code is sometimes uncertain due to the retrospective design of the study, which may lead to overestimation of the disease. Notably, the FinnGen study identified disease traits with several healthcare registries (i.e., Drug Purchase and Drug Reimbursement and Digital and Population Data Services Agency, Digital and Population Data Services Agency, Statistics Finland; Register of Primary Health Care Visits, Care Register for Health Care; and Finnish Cancer Registry). Diagnosis of migraine may largely relay on physicians rather than according to the International Classification of Headache Disorders criteria by neurologists. All these may contribute to the higher proportion of MWA observed in the FinnGen study.

In conclusion, a causal relationship between genetically delayed age at first sexual intercourse and decreased risk of migraine is uncovered through our present MR analysis, which indicates the presence of extracranial regulatory pathways of migraine and needs further determination in future real-world investigations.

## Data availability statement

The original contributions presented in the study are included in the article/Supplementary material, further inquiries can be directed to the corresponding author.

## Ethics statement

This study is a secondary analysis of centralized data from public databases, and the experiments providing this data have been approved by an ethical review and the participants’ consent. The studies were conducted in accordance with the local legislation and institutional requirements. Written informed consent for participation in this study was provided by the participants’ legal guardians/next of kin.

## Author contributions

GZ: Data curation, Formal analysis, Investigation, Methodology, Project administration, Software, Visualization, Writing – original draft. MW: Data curation, Resources, Supervision, Validation, Writing – review & editing. YW: Supervision, Validation, Visualization, Writing – review & editing. FK: Conceptualization, Funding acquisition, Writing – review & editing.

## Footnotes

^1^ https://www.ebi.ac.uk/gwas/

^2^ https://r9.finngen.fi

^3^ http://www.phenoscanner.medschl.cam.ac.uk

^4^ cnsgenomics.com/
