# Supplementary material for: Assessing the association between age at first sexual intercourse and migraine: a Mendelian randomization study
Source: Front Neurol. 2024 Feb 6;15:1347831. doi: 10.3389/fneur.2024.1347831 (PMC10876792; doi:10.3389/fneur.2024.1347831)
Supplement: Supplementary file 1 [file Data_Sheet_1.docx]

Supplement Table 1: Characteristics of candidate genetic instruments (AFS)

| **SNP** | **Chr** | **pos** | **EA** | **OA** | **Beta** | **SE** | ***P* value** | **F-statistic** | **Sample size** | **Palindromic** |
| --- | --- | --- | --- | --- | --- | --- | --- | --- | --- | --- |
| rs10104523 | 8 | 73889570 | C | T | -0.0123 | 0.0021 | 6.70E-10 | 34.31 | 397338 | FALSE |
| rs10144067 | 14 | 93885198 | T | C | -0.0131 | 0.0021 | 1.60E-09 | 38.91 | 397338 | FALSE |
| rs10469020 | 18 | 50811573 | T | A | 0.0217 | 0.0038 | 1.50E-08 | 32.61 | 397338 | TRUE |
| rs10516875 | 4 | 91590266 | G | T | -0.0148 | 0.0024 | 6.60E-10 | 38.03 | 397338 | FALSE |
| rs10746578 | 9 | 81512820 | G | A | 0.0129 | 0.0022 | 5.40E-09 | 34.38 | 397338 | FALSE |
| rs10749233 | 10 | 1.19E+08 | C | G | 0.0175 | 0.0024 | 9.20E-13 | 53.17 | 397338 | TRUE |
| rs10853981 | 19 | 4965064 | A | G | -0.0123 | 0.0022 | 1.00E-08 | 31.26 | 397338 | FALSE |
| rs10858054 | 1 | 1.15E+08 | T | G | 0.0149 | 0.0027 | 2.30E-08 | 30.45 | 397338 | FALSE |
| rs10871582 | 18 | 53276589 | T | G | -0.0153 | 0.0022 | 1.30E-12 | 48.37 | 397338 | FALSE |
| rs10880086 | 12 | 41905017 | A | G | 0.016 | 0.0025 | 1.30E-10 | 40.96 | 397338 | FALSE |
| rs10922907 | 1 | 91193049 | T | A | 0.0211 | 0.0021 | 2.10E-24 | 100.95 | 397338 | TRUE |
| rs10955084 | 8 | 97825208 | T | C | -0.0131 | 0.0021 | 2.50E-10 | 38.91 | 397338 | FALSE |
| rs10978435 | 9 | 1.09E+08 | C | T | -0.0151 | 0.0022 | 1.20E-11 | 47.11 | 397338 | FALSE |
| rs10992812 | 9 | 96392182 | A | G | 0.0121 | 0.0022 | 1.70E-08 | 30.25 | 397338 | FALSE |
| rs11240331 | 1 | 2.05E+08 | T | C | 0.014 | 0.0024 | 2.20E-09 | 34.03 | 397338 | FALSE |
| rs112633616 | 12 | 23238326 | C | A | 0.0386 | 0.0058 | 5.20E-11 | 44.29 | 397338 | FALSE |
| rs113247159 | 7 | 1.4E+08 | C | T | 0.0156 | 0.0024 | 1.10E-10 | 42.25 | 397338 | FALSE |
| rs113338260 | 5 | 46004640 | C | T | -0.0149 | 0.0025 | 2.20E-09 | 35.52 | 397338 | FALSE |
| rs1156981 | 1 | 88829969 | G | A | -0.0207 | 0.0035 | 1.60E-09 | 34.98 | 397338 | FALSE |
| rs11678980 | 2 | 1.62E+08 | A | G | -0.012 | 0.0021 | 1.20E-08 | 32.65 | 397338 | FALSE |
| rs11688027 | 2 | 78018164 | G | A | 0.0224 | 0.004 | 1.40E-08 | 31.36 | 397338 | FALSE |
| rs11729080 | 4 | 1.13E+08 | A | G | 0.0222 | 0.0027 | 4.10E-16 | 67.6 | 397338 | FALSE |
| rs117831144 | 20 | 14731057 | T | C | -0.0356 | 0.0064 | 3.40E-08 | 30.94 | 397338 | FALSE |
| rs11866420 | 16 | 90054704 | G | C | -0.0168 | 0.0021 | 7.60E-16 | 64 | 397338 | TRUE |
| rs12049116 | 1 | 1.51E+08 | T | C | 0.0151 | 0.0025 | 1.90E-09 | 36.48 | 397338 | FALSE |
| rs12147463 | 14 | 41059928 | A | G | -0.0202 | 0.0026 | 3.60E-14 | 60.36 | 397338 | FALSE |
| rs12203592 | 6 | 396321 | T | C | 0.014 | 0.0026 | 3.60E-08 | 28.99 | 397338 | FALSE |
| rs12204714 | 6 | 1.52E+08 | T | C | 0.0275 | 0.0021 | 8.30E-38 | 171.49 | 397338 | FALSE |
| rs1226414 | 2 | 1.57E+08 | T | A | 0.0144 | 0.0021 | 2.30E-12 | 47.02 | 397338 | TRUE |
| rs12292980 | 11 | 27734349 | A | G | -0.0127 | 0.0022 | 1.10E-08 | 33.32 | 397338 | FALSE |
| rs12376530 | 9 | 14529836 | C | A | -0.0145 | 0.0024 | 7.90E-10 | 36.5 | 397338 | FALSE |
| rs12448731 | 16 | 49622284 | T | C | -0.0179 | 0.003 | 2.10E-09 | 35.6 | 397338 | FALSE |
| rs12463727 | 2 | 26948413 | A | G | 0.0141 | 0.0021 | 5.90E-12 | 45.08 | 397338 | FALSE |
| rs12511982 | 4 | 60736871 | A | G | 0.0115 | 0.0021 | 1.60E-08 | 29.99 | 397338 | FALSE |
| rs12523097 | 5 | 1.67E+08 | C | T | -0.0138 | 0.0023 | 1.20E-09 | 36 | 397338 | FALSE |
| rs12523398 | 5 | 45119647 | A | T | 0.0219 | 0.0027 | 1.10E-15 | 65.79 | 397338 | TRUE |
| rs12554512 | 9 | 23352293 | C | T | 0.0126 | 0.0021 | 1.20E-09 | 36 | 397338 | FALSE |
| rs12653396 | 5 | 87847273 | A | T | -0.0199 | 0.0021 | 2.00E-21 | 89.8 | 397338 | TRUE |
| rs12714702 | 3 | 88249922 | G | A | -0.0242 | 0.0028 | 1.40E-17 | 74.7 | 397338 | FALSE |
| rs12757779 | 1 | 2.33E+08 | A | G | 0.0134 | 0.0025 | 3.70E-08 | 28.73 | 397338 | FALSE |
| rs12907546 | 15 | 47684280 | A | G | -0.0227 | 0.0025 | 2.10E-19 | 82.45 | 397338 | FALSE |
| rs13175535 | 5 | 31078814 | A | G | 0.0125 | 0.0023 | 4.40E-08 | 29.54 | 397338 | FALSE |
| rs13178956 | 5 | 1.55E+08 | T | A | -0.0164 | 0.0024 | 4.20E-12 | 46.69 | 397338 | TRUE |
| rs1320138 | 2 | 1.44E+08 | C | T | 0.0148 | 0.0021 | 1.90E-12 | 49.67 | 397338 | FALSE |
| rs1320330 | 2 | 622225 | G | T | -0.0178 | 0.0027 | 1.10E-10 | 43.46 | 397338 | FALSE |
| rs13307225 | 7 | 1.05E+08 | A | G | 0.0218 | 0.0034 | 4.70E-11 | 41.11 | 397338 | FALSE |
| rs1368546 | 2 | 1.04E+08 | C | T | 0.0165 | 0.0021 | 2.50E-15 | 61.73 | 397338 | FALSE |
| rs1392816 | 1 | 66481188 | T | C | 0.017 | 0.0021 | 6.20E-16 | 65.53 | 397338 | FALSE |
| rs141547796 | 6 | 50615935 | A | G | 0.0324 | 0.0038 | 1.40E-17 | 72.7 | 397338 | FALSE |
| rs147633738 | 1 | 1.56E+08 | T | C | -0.0242 | 0.0042 | 8.10E-09 | 33.2 | 397338 | FALSE |
| rs147725178 | 2 | 1.86E+08 | T | C | -0.0307 | 0.0054 | 5.70E-09 | 32.32 | 397338 | FALSE |
| rs1585634 | 8 | 54396376 | C | G | -0.0146 | 0.0026 | 1.30E-08 | 31.53 | 397338 | TRUE |
| rs1609598 | 20 | 51510926 | T | C | -0.0122 | 0.0022 | 3.30E-08 | 30.75 | 397338 | FALSE |
| rs1866710 | 11 | 12875312 | G | A | 0.0148 | 0.0023 | 2.30E-10 | 41.41 | 397338 | FALSE |
| rs1925686 | 6 | 87858691 | A | G | 0.0123 | 0.0021 | 7.30E-09 | 34.31 | 397338 | FALSE |
| rs1931263 | 1 | 96175101 | T | G | -0.0117 | 0.0021 | 1.70E-08 | 31.04 | 397338 | FALSE |
| rs1991651 | 8 | 10706411 | G | C | 0.0182 | 0.0021 | 1.70E-17 | 75.11 | 397338 | TRUE |
| rs1995181 | 12 | 24195048 | A | T | -0.0121 | 0.0021 | 4.50E-09 | 33.2 | 397338 | TRUE |
| rs2084572 | 3 | 17315758 | G | A | 0.014 | 0.0021 | 3.50E-11 | 44.44 | 397338 | FALSE |
| rs2091377 | 2 | 6145158 | T | C | 0.0118 | 0.0021 | 3.10E-08 | 31.57 | 397338 | FALSE |
| rs2093623 | 10 | 10922977 | A | G | 0.0139 | 0.0021 | 4.50E-11 | 43.81 | 397338 | FALSE |
| rs2130894 | 10 | 1.34E+08 | T | C | 0.0131 | 0.0024 | 1.70E-08 | 29.79 | 397338 | FALSE |
| rs215639 | 7 | 32373639 | T | C | 0.0131 | 0.0022 | 2.30E-09 | 35.46 | 397338 | FALSE |
| rs2174752 | 13 | 69332015 | T | G | -0.0128 | 0.0021 | 5.90E-10 | 37.15 | 397338 | FALSE |
| rs2176337 | 9 | 1.09E+08 | T | A | -0.0151 | 0.0022 | 1.10E-11 | 47.11 | 397338 | TRUE |
| rs2188151 | 3 | 50201924 | T | G | -0.021 | 0.0021 | 2.10E-24 | 100 | 397338 | FALSE |
| rs222440 | 6 | 52946320 | C | T | 0.017 | 0.0027 | 4.90E-10 | 39.64 | 397338 | FALSE |
| rs2274568 | 1 | 1.11E+08 | A | G | -0.0129 | 0.0021 | 4.60E-10 | 37.73 | 397338 | FALSE |
| rs2279574 | 12 | 89745477 | A | C | 0.0119 | 0.0021 | 2.60E-08 | 32.11 | 397338 | FALSE |
| rs2406374 | 5 | 1.07E+08 | T | C | 0.0139 | 0.0022 | 4.30E-10 | 39.92 | 397338 | FALSE |
| rs2612030 | 3 | 53773437 | C | T | 0.024 | 0.0028 | 1.20E-17 | 73.47 | 397338 | FALSE |
| rs2650705 | 10 | 63239803 | G | A | -0.0166 | 0.0028 | 3.70E-09 | 35.15 | 397338 | FALSE |
| rs28406364 | 17 | 47454507 | T | C | -0.0165 | 0.0021 | 8.10E-15 | 61.73 | 397338 | FALSE |
| rs28929474 | 14 | 94844947 | T | C | 0.0416 | 0.0074 | 1.20E-08 | 31.6 | 397338 | FALSE |
| rs2974311 | 8 | 42455166 | A | G | 0.0131 | 0.0021 | 2.70E-10 | 38.91 | 397338 | FALSE |
| rs3007104 | 14 | 47367434 | A | G | -0.0148 | 0.0021 | 1.50E-12 | 49.67 | 397338 | FALSE |
| rs341521 | 13 | 60399045 | A | G | -0.0159 | 0.0023 | 9.00E-13 | 47.79 | 397338 | FALSE |
| rs3447 | 9 | 86327243 | G | C | 0.0177 | 0.0029 | 3.70E-09 | 37.25 | 397338 | TRUE |
| rs34517439 | 1 | 78450517 | A | C | -0.0195 | 0.0032 | 8.20E-10 | 37.13 | 397338 | FALSE |
| rs34606772 | 3 | 24908376 | T | C | -0.0118 | 0.0021 | 1.60E-08 | 31.57 | 397338 | FALSE |
| rs34804222 | 11 | 43771084 | G | A | 0.0151 | 0.0021 | 1.90E-13 | 51.7 | 397338 | FALSE |
| rs34811474 | 4 | 25408838 | A | G | 0.0155 | 0.0024 | 1.30E-10 | 41.71 | 397338 | FALSE |
| rs35077383 | 1 | 1.52E+08 | C | T | -0.011 | 0.0021 | 3.90E-08 | 27.44 | 397338 | FALSE |
| rs35851551 | 7 | 31330785 | G | A | -0.0247 | 0.0034 | 2.50E-13 | 52.78 | 397338 | FALSE |
| rs359271 | 2 | 60463149 | C | T | -0.0181 | 0.0021 | 4.10E-18 | 74.29 | 397338 | FALSE |
| rs3896224 | 10 | 1.06E+08 | G | A | 0.0198 | 0.0021 | 2.70E-21 | 88.9 | 397338 | FALSE |
| rs410520 | 17 | 4938924 | T | C | 0.0113 | 0.0021 | 4.60E-08 | 28.95 | 397338 | FALSE |
| rs4246175 | 9 | 1.35E+08 | A | T | 0.0144 | 0.0022 | 4.20E-11 | 42.84 | 397338 | TRUE |
| rs4439537 | 11 | 79887549 | C | T | 0.0132 | 0.0021 | 1.10E-10 | 39.51 | 397338 | FALSE |
| rs4557006 | 2 | 22443840 | A | G | -0.0118 | 0.0021 | 8.30E-09 | 31.57 | 397338 | FALSE |
| rs4569188 | 14 | 93915929 | A | G | -0.0135 | 0.0022 | 1.30E-09 | 37.65 | 397338 | FALSE |
| rs4602427 | 3 | 1.17E+08 | G | C | -0.0172 | 0.0026 | 2.30E-11 | 43.76 | 397338 | TRUE |
| rs4702 | 15 | 91426560 | A | G | 0.0172 | 0.0021 | 1.10E-16 | 67.08 | 397338 | FALSE |
| rs4709807 | 6 | 1.64E+08 | C | T | -0.0138 | 0.0024 | 1.00E-08 | 33.06 | 397338 | FALSE |
| rs4800204 | 18 | 22647270 | T | C | -0.0125 | 0.0021 | 4.60E-09 | 35.43 | 397338 | FALSE |
| rs4809230 | 20 | 62439274 | A | G | 0.0231 | 0.0039 | 2.50E-09 | 35.08 | 397338 | FALSE |
| rs4868800 | 5 | 1.67E+08 | T | G | 0.0128 | 0.0021 | 8.20E-10 | 37.15 | 397338 | FALSE |
| rs4952343 | 2 | 32858637 | G | A | 0.0126 | 0.0021 | 2.20E-09 | 36 | 397338 | FALSE |
| rs4961705 | 9 | 16347927 | C | G | 0.013 | 0.0022 | 4.20E-09 | 34.92 | 397338 | TRUE |
| rs56306056 | 2 | 1.84E+08 | A | G | 0.0157 | 0.0025 | 4.10E-10 | 39.44 | 397338 | FALSE |
| rs56392241 | 3 | 1.32E+08 | C | A | -0.014 | 0.0021 | 8.90E-12 | 44.44 | 397338 | FALSE |
| rs58938116 | 7 | 1.22E+08 | T | G | -0.0175 | 0.0028 | 1.60E-10 | 39.06 | 397338 | FALSE |
| rs590414 | 11 | 1.06E+08 | T | A | 0.0135 | 0.0021 | 1.70E-10 | 41.33 | 397338 | TRUE |
| rs592278 | 18 | 40231833 | A | G | -0.0125 | 0.0021 | 3.40E-09 | 35.43 | 397338 | FALSE |
| rs6058613 | 20 | 30864279 | G | C | -0.0171 | 0.0028 | 1.80E-09 | 37.3 | 397338 | TRUE |
| rs60775983 | 5 | 1.24E+08 | G | A | 0.0178 | 0.0029 | 4.50E-10 | 37.67 | 397338 | FALSE |
| rs61856978 | 10 | 97941022 | C | T | -0.0154 | 0.0022 | 5.70E-13 | 49 | 397338 | FALSE |
| rs62134195 | 2 | 45062249 | T | C | 0.0318 | 0.0052 | 5.30E-10 | 37.4 | 397338 | FALSE |
| rs62177795 | 2 | 63475640 | A | G | 0.019 | 0.0025 | 9.00E-15 | 57.76 | 397338 | FALSE |
| rs62439690 | 7 | 21417556 | A | G | -0.0155 | 0.0024 | 7.20E-11 | 41.71 | 397338 | FALSE |
| rs6517512 | 21 | 40512129 | G | A | -0.0275 | 0.005 | 3.20E-08 | 30.25 | 397338 | FALSE |
| rs6564268 | 16 | 75606878 | G | C | 0.0266 | 0.0045 | 5.50E-09 | 34.94 | 397338 | TRUE |
| rs6586405 | 1 | 2.35E+08 | A | C | -0.0122 | 0.0022 | 3.00E-08 | 30.75 | 397338 | FALSE |
| rs6719762 | 2 | 60166832 | C | T | -0.0221 | 0.0021 | 7.20E-27 | 110.75 | 397338 | FALSE |
| rs6747099 | 2 | 60777498 | C | G | 0.0123 | 0.0021 | 4.90E-09 | 34.31 | 397338 | TRUE |
| rs6748341 | 2 | 2.25E+08 | G | C | 0.0149 | 0.0022 | 1.10E-11 | 45.87 | 397338 | TRUE |
| rs67723420 | 3 | 35775115 | A | T | 0.0146 | 0.0021 | 1.30E-11 | 48.34 | 397338 | TRUE |
| rs6776937 | 3 | 60870307 | A | C | -0.0138 | 0.0025 | 1.20E-08 | 30.47 | 397338 | FALSE |
| rs6966769 | 7 | 1299334 | G | A | 0.0182 | 0.0033 | 1.90E-08 | 30.42 | 397338 | FALSE |
| rs6978112 | 7 | 1966841 | T | C | -0.0158 | 0.0021 | 2.70E-14 | 56.61 | 397338 | FALSE |
| rs7008955 | 8 | 26334103 | G | T | 0.0116 | 0.0021 | 3.40E-08 | 30.51 | 397338 | FALSE |
| rs702 | 4 | 28710551 | T | A | 0.0181 | 0.0028 | 2.80E-10 | 41.79 | 397338 | TRUE |
| rs705240 | 3 | 1.18E+08 | T | C | -0.015 | 0.0027 | 7.00E-09 | 30.86 | 397338 | FALSE |
| rs7079070 | 10 | 1.34E+08 | A | G | -0.0165 | 0.0021 | 1.20E-15 | 61.73 | 397338 | FALSE |
| rs7091634 | 10 | 9973015 | G | A | 0.0125 | 0.0021 | 3.80E-09 | 35.43 | 397338 | FALSE |
| rs7110863 | 11 | 1.13E+08 | G | A | -0.0154 | 0.0021 | 5.00E-13 | 53.78 | 397338 | FALSE |
| rs714393 | 2 | 2.13E+08 | T | C | 0.0126 | 0.0021 | 1.50E-09 | 36 | 397338 | FALSE |
| rs7151954 | 14 | 1.03E+08 | G | A | -0.0208 | 0.0026 | 4.00E-16 | 64 | 397338 | FALSE |
| rs7152323 | 14 | 98553482 | G | A | -0.0156 | 0.0021 | 3.10E-13 | 55.18 | 397338 | FALSE |
| rs7167444 | 15 | 97495941 | T | G | -0.0138 | 0.0024 | 8.10E-09 | 33.06 | 397338 | FALSE |
| rs7188873 | 16 | 24727064 | G | A | -0.0146 | 0.0021 | 5.80E-12 | 48.34 | 397338 | FALSE |
| rs7201310 | 16 | 12513797 | A | C | 0.0121 | 0.0023 | 2.70E-08 | 27.68 | 397338 | FALSE |
| rs7236339 | 18 | 77579773 | A | G | -0.0201 | 0.0025 | 3.90E-16 | 64.64 | 397338 | FALSE |
| rs72674824 | 8 | 95489281 | C | T | 0.0137 | 0.0024 | 3.40E-08 | 32.59 | 397338 | FALSE |
| rs72887338 | 6 | 67536056 | C | T | -0.0163 | 0.0021 | 1.60E-14 | 60.25 | 397338 | FALSE |
| rs7381195 | 5 | 60030791 | A | T | -0.0136 | 0.0021 | 1.60E-10 | 41.94 | 397338 | TRUE |
| rs7452074 | 6 | 1.25E+08 | C | A | -0.0128 | 0.0021 | 4.90E-10 | 37.15 | 397338 | FALSE |
| rs74737734 | 14 | 30726670 | T | A | 0.0353 | 0.0064 | 2.70E-08 | 30.42 | 397338 | TRUE |
| rs7476 | 11 | 46342834 | C | A | -0.014 | 0.0022 | 5.60E-10 | 40.5 | 397338 | FALSE |
| rs7503604 | 17 | 79095629 | A | C | 0.0129 | 0.0021 | 6.10E-10 | 37.73 | 397338 | FALSE |
| rs7525548 | 1 | 75001474 | T | A | 0.0145 | 0.0021 | 2.90E-12 | 47.68 | 397338 | TRUE |
| rs7566527 | 2 | 1.01E+08 | T | C | 0.0137 | 0.0021 | 3.80E-11 | 42.56 | 397338 | FALSE |
| rs7575189 | 2 | 1.74E+08 | A | G | 0.0164 | 0.0021 | 1.90E-15 | 60.99 | 397338 | FALSE |
| rs7618715 | 3 | 70873278 | A | G | 0.0126 | 0.0021 | 1.90E-09 | 36 | 397338 | FALSE |
| rs763053 | 16 | 735921 | C | T | 0.0163 | 0.0025 | 4.90E-11 | 42.51 | 397338 | FALSE |
| rs76513770 | 16 | 72505534 | C | T | 0.024 | 0.0031 | 4.90E-15 | 59.94 | 397338 | FALSE |
| rs766406 | 6 | 26319588 | T | G | 0.0135 | 0.0021 | 5.20E-10 | 41.33 | 397338 | FALSE |
| rs7671317 | 4 | 62972597 | T | G | 0.0122 | 0.0022 | 3.50E-08 | 30.75 | 397338 | FALSE |
| rs767943 | 6 | 23446691 | A | C | -0.0171 | 0.0024 | 5.70E-13 | 50.77 | 397338 | FALSE |
| rs7704530 | 5 | 30845465 | A | G | -0.0128 | 0.0023 | 2.90E-08 | 30.97 | 397338 | FALSE |
| rs7783012 | 7 | 1.14E+08 | A | G | -0.0196 | 0.0021 | 1.40E-20 | 87.11 | 397338 | FALSE |
| rs7785195 | 7 | 3424686 | A | G | 0.0142 | 0.0022 | 9.50E-11 | 41.66 | 397338 | FALSE |
| rs7804551 | 7 | 99119110 | G | A | 0.0226 | 0.0029 | 2.90E-15 | 60.73 | 397338 | FALSE |
| rs7815125 | 8 | 87680112 | A | T | 0.0179 | 0.0027 | 3.30E-11 | 43.95 | 397338 | TRUE |
| rs7824756 | 8 | 51118559 | C | T | -0.0155 | 0.0023 | 2.80E-11 | 45.42 | 397338 | FALSE |
| rs783544 | 15 | 83240293 | C | A | 0.0148 | 0.0024 | 3.50E-10 | 38.03 | 397338 | FALSE |
| rs7909331 | 10 | 11205224 | G | A | -0.0156 | 0.0028 | 2.90E-08 | 31.04 | 397338 | FALSE |
| rs79269403 | 3 | 1.08E+08 | A | G | 0.0206 | 0.0025 | 3.10E-17 | 67.9 | 397338 | FALSE |
| rs7927195 | 11 | 1.27E+08 | G | A | -0.0152 | 0.0021 | 4.90E-13 | 52.39 | 397338 | FALSE |
| rs7942078 | 11 | 28656064 | T | A | 0.0161 | 0.0022 | 6.60E-14 | 53.56 | 397338 | TRUE |
| rs794375 | 7 | 75147801 | C | T | 0.0142 | 0.0021 | 1.40E-11 | 45.72 | 397338 | FALSE |
| rs7955865 | 12 | 56468706 | T | A | -0.0134 | 0.0022 | 8.40E-10 | 37.1 | 397338 | TRUE |
| rs7972441 | 12 | 84043146 | A | C | -0.0149 | 0.0022 | 1.10E-11 | 45.87 | 397338 | FALSE |
| rs803679 | 1 | 44349405 | A | G | 0.0175 | 0.0025 | 6.80E-12 | 49 | 397338 | FALSE |
| rs807478 | 19 | 36252494 | G | A | 0.0122 | 0.0021 | 1.90E-09 | 33.75 | 397338 | FALSE |
| rs809955 | 4 | 1.41E+08 | A | G | 0.0157 | 0.0021 | 1.00E-13 | 55.89 | 397338 | FALSE |
| rs8180995 | 8 | 1.43E+08 | G | A | 0.0122 | 0.0021 | 8.90E-10 | 33.75 | 397338 | FALSE |
| rs838042 | 2 | 1.4E+08 | A | G | 0.0162 | 0.0022 | 3.90E-13 | 54.22 | 397338 | FALSE |
| rs9403187 | 6 | 1E+08 | T | A | 0.0149 | 0.0022 | 1.70E-11 | 45.87 | 397338 | TRUE |
| rs9514600 | 13 | 1.08E+08 | G | C | -0.0114 | 0.0021 | 2.40E-08 | 29.47 | 397338 | TRUE |
| rs9538248 | 13 | 59492828 | A | C | -0.0159 | 0.0022 | 3.00E-13 | 52.23 | 397338 | FALSE |
| rs9809849 | 3 | 3726156 | A | G | -0.014 | 0.0021 | 1.80E-11 | 44.44 | 397338 | FALSE |
| rs9835772 | 3 | 85766025 | T | A | -0.0138 | 0.0024 | 9.90E-09 | 33.06 | 397338 | TRUE |
| rs9923553 | 16 | 5825579 | G | A | -0.0139 | 0.0023 | 3.80E-10 | 36.52 | 397338 | FALSE |
| rs993700 | 4 | 67825894 | C | T | 0.0157 | 0.0025 | 2.90E-10 | 39.44 | 397338 | FALSE |

**Abbreviations:** EA, effect allele; OA other allele; SNP, single nucleotide polymorphism; AFS, age at first sexual intercourse.

Supplement Table 2: Characteristics of candidate genetic instruments (AFSM)

| **SNP** | **Chr** | **pos** | **EA** | **OA** | **Beta** | **SE** | ***P* value** | **F-statistic** | **Sample size** | **Palindromic** |
| --- | --- | --- | --- | --- | --- | --- | --- | --- | --- | --- |
| rs10978435 | 9 | 1.09E+08 | C | T | -0.0229 | 0.0033 | 8.50E-12 | 48.15519 | 182791 | FALSE |
| rs11038866 | 11 | 46355932 | G | C | -0.0267 | 0.0034 | 8.90E-15 | 61.66869 | 182791 | TRUE |
| rs11155821 | 6 | 1.52E+08 | C | T | 0.0263 | 0.0032 | 1.60E-16 | 67.54785 | 182791 | FALSE |
| rs11160699 | 14 | 1.03E+08 | A | G | -0.0251 | 0.0043 | 1.50E-09 | 34.07301 | 182791 | FALSE |
| rs11231967 | 11 | 79898870 | A | C | -0.0184 | 0.0033 | 1.90E-08 | 31.08907 | 182791 | FALSE |
| rs11729080 | 4 | 1.13E+08 | A | G | 0.0236 | 0.0041 | 8.40E-09 | 33.13266 | 182791 | FALSE |
| rs11866420 | 16 | 90054704 | G | C | -0.0239 | 0.0031 | 5.00E-14 | 59.43913 | 182791 | TRUE |
| rs12042107 | 1 | 91196176 | C | T | 0.0226 | 0.0031 | 5.50E-13 | 53.1488 | 182791 | FALSE |
| rs12369701 | 12 | 31674691 | T | G | -0.0171 | 0.0031 | 3.80E-08 | 30.42768 | 182791 | FALSE |
| rs12523398 | 5 | 45119647 | A | T | 0.028 | 0.0041 | 2.00E-11 | 46.63891 | 182791 | TRUE |
| rs12653396 | 5 | 87847273 | A | T | -0.0189 | 0.0032 | 8.10E-10 | 34.88379 | 182791 | TRUE |
| rs12664423 | 6 | 1.01E+08 | G | T | -0.0196 | 0.0036 | 3.70E-08 | 29.64198 | 182791 | FALSE |
| rs12707087 | 7 | 1.33E+08 | C | T | 0.0259 | 0.0039 | 2.40E-11 | 44.10322 | 182791 | FALSE |
| rs12736932 | 1 | 2.33E+08 | A | G | 0.02 | 0.0037 | 4.00E-08 | 29.21841 | 182791 | FALSE |
| rs1275939 | 2 | 26957737 | A | G | -0.0198 | 0.0031 | 1.70E-10 | 40.79501 | 182791 | FALSE |
| rs12900091 | 15 | 47886897 | G | A | 0.0238 | 0.0031 | 2.00E-14 | 58.94277 | 182791 | FALSE |
| rs13251378 | 8 | 42468218 | T | C | 0.0171 | 0.0032 | 4.80E-08 | 28.55566 | 182791 | FALSE |
| rs13261725 | 8 | 91866297 | C | G | -0.0203 | 0.0035 | 1.40E-08 | 33.64 | 182791 | TRUE |
| rs1392816 | 1 | 66481188 | T | C | 0.0196 | 0.0032 | 6.80E-10 | 37.51563 | 182791 | FALSE |
| rs141547796 | 6 | 50615935 | A | G | 0.0394 | 0.0057 | 3.60E-12 | 47.77962 | 182791 | FALSE |
| rs1561029 | 3 | 1.08E+08 | C | A | -0.0199 | 0.0032 | 3.00E-10 | 38.67285 | 182791 | FALSE |
| rs2612032 | 3 | 53768915 | G | A | 0.029 | 0.0043 | 1.20E-11 | 45.48405 | 182791 | FALSE |
| rs2624839 | 3 | 50202231 | C | T | -0.025 | 0.0031 | 1.10E-15 | 65.03642 | 182791 | FALSE |
| rs266070 | 2 | 1.04E+08 | G | A | 0.0208 | 0.0031 | 1.40E-11 | 45.01977 | 182791 | FALSE |
| rs28483459 | 16 | 71998398 | T | C | 0.021 | 0.0034 | 3.70E-10 | 38.14879 | 182791 | FALSE |
| rs28808991 | 15 | 83260979 | C | G | 0.0182 | 0.0032 | 7.40E-09 | 32.34766 | 182791 | TRUE |
| rs292062 | 4 | 28499782 | G | A | -0.0204 | 0.0035 | 5.70E-09 | 33.97224 | 182791 | FALSE |
| rs340218 | 13 | 60442830 | G | T | -0.0205 | 0.0034 | 1.20E-09 | 36.35381 | 182791 | FALSE |
| rs34481141 | 2 | 1.86E+08 | G | A | 0.0253 | 0.0043 | 7.20E-09 | 34.61817 | 182791 | FALSE |
| rs359233 | 2 | 60470926 | G | A | -0.0235 | 0.0032 | 2.00E-13 | 53.93066 | 182791 | FALSE |
| rs3758790 | 11 | 1.06E+08 | G | A | 0.0213 | 0.0032 | 4.90E-11 | 44.30566 | 182791 | FALSE |
| rs3768470 | 1 | 1.11E+08 | G | A | -0.0172 | 0.0031 | 4.00E-08 | 30.7846 | 182791 | FALSE |
| rs3896224 | 10 | 1.06E+08 | G | A | 0.0189 | 0.0032 | 2.20E-09 | 34.88379 | 182791 | FALSE |
| rs4687959 | 3 | 1.19E+08 | C | T | 0.0204 | 0.0036 | 1.10E-08 | 32.11111 | 182791 | FALSE |
| rs4702 | 15 | 91426560 | A | G | 0.0213 | 0.0031 | 7.10E-12 | 47.2102 | 182791 | FALSE |
| rs4958410 | 5 | 1.55E+08 | T | C | -0.0225 | 0.0037 | 1.20E-09 | 36.97955 | 182791 | FALSE |
| rs6087358 | 20 | 30855746 | G | A | -0.0253 | 0.0045 | 1.60E-08 | 31.60938 | 182791 | FALSE |
| rs616827 | 1 | 75703209 | T | G | -0.0194 | 0.0035 | 4.30E-08 | 30.72327 | 182791 | FALSE |
| rs61856978 | 10 | 97941022 | C | T | -0.0202 | 0.0033 | 2.50E-10 | 37.46924 | 182791 | FALSE |
| rs62370848 | 5 | 1.24E+08 | C | A | 0.0223 | 0.0039 | 1.20E-08 | 32.69494 | 182791 | FALSE |
| rs6508144 | 18 | 50026142 | G | C | 0.0168 | 0.0031 | 3.80E-08 | 29.36941 | 182791 | TRUE |
| rs6974757 | 7 | 1.14E+08 | G | C | -0.0235 | 0.0032 | 1.20E-13 | 53.93066 | 182791 | TRUE |
| rs7024334 | 9 | 1.09E+08 | G | T | 0.0286 | 0.0037 | 4.10E-14 | 59.74872 | 182791 | FALSE |
| rs7111153 | 11 | 1.13E+08 | C | T | -0.019 | 0.0032 | 3.00E-09 | 35.25391 | 182791 | FALSE |
| rs7188873 | 16 | 24727064 | G | A | -0.019 | 0.0032 | 4.10E-09 | 35.25391 | 182791 | FALSE |
| rs7236339 | 18 | 77579773 | A | G | -0.0224 | 0.0037 | 2.40E-09 | 36.65157 | 182791 | FALSE |
| rs72863829 | 10 | 1.34E+08 | T | C | -0.0261 | 0.0039 | 3.40E-11 | 44.78698 | 182791 | FALSE |
| rs7297175 | 12 | 56473808 | C | T | -0.0183 | 0.0031 | 3.30E-09 | 34.84807 | 182791 | FALSE |
| rs72990858 | 6 | 1.05E+08 | A | G | 0.0341 | 0.0053 | 1.50E-10 | 41.39587 | 182791 | FALSE |
| rs73029605 | 3 | 15432809 | T | C | 0.051 | 0.009 | 2.10E-08 | 32.11111 | 182791 | FALSE |
| rs75120545 | 2 | 44271496 | T | C | 0.0714 | 0.0095 | 7.40E-14 | 56.48709 | 182791 | FALSE |
| rs7598402 | 2 | 50735943 | G | C | 0.0174 | 0.0031 | 2.00E-08 | 31.50468 | 182791 | TRUE |
| rs76702070 | 11 | 85398919 | A | T | 0.0469 | 0.0077 | 1.30E-09 | 37.09917 | 182791 | TRUE |
| rs78945375 | 8 | 8731567 | G | C | 0.0296 | 0.004 | 1.30E-13 | 54.76 | 182791 | TRUE |
| rs794375 | 7 | 75147801 | C | T | 0.0203 | 0.0032 | 1.20E-10 | 40.24316 | 182791 | FALSE |
| rs803679 | 1 | 44349405 | A | G | 0.0216 | 0.0038 | 1.10E-08 | 32.31025 | 182791 | FALSE |
| rs9351589 | 6 | 67405215 | T | G | -0.0205 | 0.0033 | 3.10E-10 | 38.59045 | 182791 | FALSE |
| rs960986 | 3 | 85519305 | T | C | 0.0294 | 0.0032 | 2.90E-20 | 84.41016 | 182791 | FALSE |
| rs9809849 | 3 | 3726156 | A | G | -0.0193 | 0.0031 | 8.90E-10 | 38.76067 | 182791 | FALSE |
| rs9829549 | 3 | 1.17E+08 | G | A | -0.0233 | 0.0039 | 2.10E-09 | 35.69297 | 182791 | FALSE |

**Abbreviations:** EA, effect allele; OA other allele; SNP, single nucleotide polymorphism; AFSM, age at first sexual intercourse of male.

Supplement Table 3: Characteristics of candidate genetic instruments (AFSF)

| **SNP** | **Chr** | **pos** | **EA** | **OA** | **Beta** | **SE** | ***P* value** | **F-statistic** | **Sample size** | **Palindromic** |
| --- | --- | --- | --- | --- | --- | --- | --- | --- | --- | --- |
| rs10219714 | 12 | 19119135 | T | C | 0.0157 | 0.0029 | 3.00E-08 | 29.30916 | 214547 | FALSE |
| rs10260121 | 7 | 1.15E+08 | C | T | -0.0251 | 0.0043 | 5.50E-09 | 34.07301 | 214547 | FALSE |
| rs10510025 | 10 | 1.19E+08 | T | C | -0.0205 | 0.0032 | 4.30E-10 | 41.04004 | 214547 | FALSE |
| rs10761784 | 10 | 65308750 | T | A | 0.0152 | 0.0028 | 3.30E-08 | 29.46939 | 214547 | TRUE |
| rs11037662 | 11 | 43851308 | T | G | 0.0248 | 0.004 | 3.90E-10 | 38.44 | 214547 | FALSE |
| rs11076964 | 16 | 5816678 | G | C | -0.0186 | 0.0034 | 2.40E-08 | 29.92734 | 214547 | TRUE |
| rs11131357 | 4 | 62966257 | T | C | 0.0164 | 0.003 | 4.10E-08 | 29.88444 | 214547 | FALSE |
| rs11214488 | 11 | 1.13E+08 | T | C | -0.0204 | 0.0036 | 8.60E-09 | 32.11111 | 214547 | FALSE |
| rs11240331 | 1 | 2.05E+08 | T | C | 0.0185 | 0.0032 | 5.10E-09 | 33.42285 | 214547 | FALSE |
| rs113367286 | 7 | 1.4E+08 | T | C | 0.0172 | 0.0031 | 1.20E-08 | 30.7846 | 214547 | FALSE |
| rs1157072 | 1 | 72634912 | G | A | 0.036 | 0.0063 | 9.60E-09 | 32.65306 | 214547 | FALSE |
| rs11627661 | 14 | 79556618 | C | G | -0.017 | 0.0029 | 2.70E-09 | 34.36385 | 214547 | TRUE |
| rs11656471 | 17 | 32899382 | T | G | -0.0197 | 0.0036 | 1.30E-08 | 29.94522 | 214547 | FALSE |
| rs11678980 | 2 | 1.62E+08 | A | G | -0.0156 | 0.0029 | 3.30E-08 | 28.93698 | 214547 | FALSE |
| rs12089815 | 1 | 91189933 | A | G | 0.0209 | 0.0028 | 7.20E-14 | 55.71556 | 214547 | FALSE |
| rs12203592 | 6 | 396321 | T | C | 0.0206 | 0.0034 | 4.60E-09 | 36.70934 | 214547 | FALSE |
| rs12204714 | 6 | 1.52E+08 | T | C | 0.0288 | 0.0029 | 6.30E-24 | 98.62545 | 214547 | FALSE |
| rs1226414 | 2 | 1.57E+08 | T | A | 0.0179 | 0.0028 | 7.10E-11 | 40.86862 | 214547 | TRUE |
| rs12528918 | 6 | 67554041 | G | T | -0.018 | 0.0028 | 1.30E-10 | 41.32653 | 214547 | FALSE |
| rs12541633 | 8 | 36706700 | T | A | 0.0156 | 0.0028 | 4.30E-08 | 31.04082 | 214547 | TRUE |
| rs12896157 | 14 | 41059664 | A | G | -0.0267 | 0.0035 | 3.30E-14 | 58.1951 | 214547 | FALSE |
| rs13005495 | 2 | 60157097 | G | T | -0.0248 | 0.0028 | 5.50E-19 | 78.44898 | 214547 | FALSE |
| rs13097782 | 3 | 35739575 | T | C | 0.0167 | 0.0028 | 2.10E-09 | 35.5727 | 214547 | FALSE |
| rs13192942 | 6 | 1.52E+08 | C | A | 0.0192 | 0.0031 | 9.80E-10 | 38.36004 | 214547 | FALSE |
| rs1372173 | 8 | 87684674 | G | A | 0.0221 | 0.0037 | 7.90E-10 | 35.67641 | 214547 | FALSE |
| rs1580173 | 3 | 1.08E+08 | A | G | -0.0177 | 0.0028 | 1.90E-10 | 39.96046 | 214547 | FALSE |
| rs16948048 | 17 | 47440466 | G | A | -0.0203 | 0.0029 | 1.30E-12 | 49 | 214547 | FALSE |
| rs17425189 | 1 | 57862949 | C | T | 0.02 | 0.0036 | 4.40E-08 | 30.8642 | 214547 | FALSE |
| rs198310 | 7 | 24191861 | T | A | 0.02 | 0.0034 | 3.50E-09 | 34.60208 | 214547 | TRUE |
| rs2152741 | 13 | 67135121 | T | C | 0.0233 | 0.0039 | 3.70E-09 | 35.69297 | 214547 | FALSE |
| rs226488 | 5 | 87896330 | C | T | -0.0224 | 0.0029 | 3.70E-15 | 59.66231 | 214547 | FALSE |
| rs2910032 | 5 | 1.53E+08 | T | C | 0.0174 | 0.0028 | 2.40E-10 | 38.61735 | 214547 | FALSE |
| rs3007104 | 14 | 47367434 | A | G | -0.0152 | 0.0028 | 4.50E-08 | 29.46939 | 214547 | FALSE |
| rs3896224 | 10 | 1.06E+08 | G | A | 0.021 | 0.0028 | 7.00E-14 | 56.25 | 214547 | FALSE |
| rs4727799 | 7 | 1.14E+08 | T | C | -0.0176 | 0.0029 | 1.50E-09 | 36.83234 | 214547 | FALSE |
| rs4768354 | 12 | 41824245 | T | C | 0.0152 | 0.0028 | 3.70E-08 | 29.46939 | 214547 | FALSE |
| rs4800092 | 18 | 36920318 | T | C | -0.0161 | 0.0028 | 1.20E-08 | 33.0625 | 214547 | FALSE |
| rs57537843 | 2 | 22558973 | A | G | -0.0159 | 0.0029 | 2.30E-08 | 30.06064 | 214547 | FALSE |
| rs57945129 | 3 | 1.18E+08 | T | C | 0.0236 | 0.0039 | 1.40E-09 | 36.61801 | 214547 | FALSE |
| rs62166484 | 2 | 1.42E+08 | A | T | -0.031 | 0.0055 | 1.10E-08 | 31.7686 | 214547 | TRUE |
| rs62435144 | 7 | 1279105 | A | G | 0.0298 | 0.005 | 1.20E-09 | 35.5216 | 214547 | FALSE |
| rs62499803 | 8 | 53175090 | G | A | -0.0199 | 0.0036 | 1.70E-08 | 30.55633 | 214547 | FALSE |
| rs62519833 | 8 | 65487989 | G | A | 0.0257 | 0.0044 | 4.30E-09 | 34.11622 | 214547 | FALSE |
| rs66906321 | 2 | 630070 | C | T | -0.0205 | 0.0037 | 4.60E-08 | 30.69759 | 214547 | FALSE |
| rs6767258 | 3 | 88182893 | A | G | -0.0248 | 0.0038 | 6.60E-11 | 42.5928 | 214547 | FALSE |
| rs6962772 | 7 | 99081730 | G | A | 0.0253 | 0.0038 | 8.40E-11 | 44.32756 | 214547 | FALSE |
| rs7079070 | 10 | 1.34E+08 | A | G | -0.0164 | 0.0028 | 4.50E-09 | 34.30612 | 214547 | FALSE |
| rs72693550 | 9 | 23735995 | A | C | -0.0215 | 0.0038 | 1.70E-08 | 32.01177 | 214547 | FALSE |
| rs72712556 | 4 | 1.41E+08 | A | G | 0.0184 | 0.003 | 3.20E-10 | 37.61778 | 214547 | FALSE |
| rs73077107 | 3 | 49671037 | A | G | 0.0331 | 0.0042 | 2.80E-15 | 62.10941 | 214547 | FALSE |
| rs7349386 | 2 | 2.14E+08 | C | T | 0.0357 | 0.0065 | 4.70E-08 | 30.16544 | 214547 | FALSE |
| rs7558132 | 2 | 1.74E+08 | A | G | -0.0209 | 0.0029 | 3.70E-13 | 51.93936 | 214547 | FALSE |
| rs7635829 | 3 | 1.47E+08 | A | G | -0.0188 | 0.0032 | 9.50E-09 | 34.51563 | 214547 | FALSE |
| rs76513770 | 16 | 72505534 | C | T | 0.0234 | 0.0041 | 8.50E-09 | 32.57347 | 214547 | FALSE |
| rs770082 | 12 | 89776485 | A | G | -0.0152 | 0.0028 | 4.80E-08 | 29.46939 | 214547 | FALSE |
| rs7815972 | 8 | 1.19E+08 | G | A | 0.0173 | 0.0029 | 4.60E-09 | 35.5874 | 214547 | FALSE |
| rs784255 | 18 | 53403228 | T | G | -0.0196 | 0.0028 | 7.60E-13 | 49 | 214547 | FALSE |
| rs79108591 | 14 | 98599967 | A | G | -0.0227 | 0.0034 | 1.40E-11 | 44.57526 | 214547 | FALSE |
| rs79155408 | 13 | 56969707 | T | C | -0.0338 | 0.0061 | 2.80E-08 | 30.7025 | 214547 | FALSE |
| rs7942078 | 11 | 28656064 | T | A | 0.0178 | 0.0029 | 1.50E-09 | 37.6742 | 214547 | TRUE |
| rs79532211 | 4 | 1.12E+08 | G | A | 0.0253 | 0.004 | 1.80E-10 | 40.00563 | 214547 | FALSE |
| rs8033799 | 15 | 47681384 | C | A | -0.0201 | 0.0034 | 4.40E-09 | 34.94896 | 214547 | FALSE |
| rs961522 | 2 | 58272502 | C | T | 0.0278 | 0.0028 | 8.60E-23 | 98.57653 | 214547 | FALSE |

**Abbreviations:** EA, effect allele; OA other allele; SNP, single nucleotide polymorphism; AFSF, age at first sexual intercourse of female.

Supplement Table 4: Results of forward Mendelian randomization

| Exposure & Outcome | Method | Beta | SE | *P* value | OR | OR_LCI95 | OR_UCI95 |
| --- | --- | --- | --- | --- | --- | --- | --- |
| AFS & Migraine | Inverse variance weighted | -0.320737 | 0.087555 | 0.000249 | 0.725614 | 0.611192 | 0.861457 |
| AFS & Migraine | Weighted median | -0.272822 | 0.110200 | 0.013298 | 0.761228 | 0.613353 | 0.944756 |
| AFS & Migraine | MR Egger | -0.383574 | 0.381540 | 0.316460 | 0.681422 | 0.322584 | 1.439426 |
| AFSM & Migraine | Inverse variance weighted | -0.34719 | 0.090377 | 0.000122 | 0.706673 | 0.591955 | 0.843623 |
| AFSM & Migraine | Weighted median | -0.41059 | 0.124238 | 0.00095 | 0.663258 | 0.519911 | 0.846127 |
| AFSM & Migraine | MR Egger | -0.2064 | 0.398101 | 0.606562 | 0.813505 | 0.372812 | 1.77513 |
| AFSF & Migraine | Inverse variance weighted | -0.30878 | 0.098347 | 0.001691 | 0.734345 | 0.605599 | 0.89046 |
| AFSF & Migraine | Weighted median | -0.40359 | 0.130482 | 0.001981 | 0.667918 | 0.517196 | 0.862564 |
| AFSF & Migraine | MR Egger | 0.180011 | 0.468636 | 0.702428 | 1.197231 | 0.477823 | 2.999777 |
| AFS & MWA | Inverse variance weighted | -0.32798 | 0.10872 | 0.002555 | 0.720377 | 0.582125 | 0.891464 |
| AFS & MWA | Weighted median | -0.51281 | 0.1533 | 0.000822 | 0.59881 | 0.443402 | 0.808688 |
| AFS & MWA | MR Egger | -0.1066 | 0.471147 | 0.821336 | 0.898888 | 0.356991 | 2.263364 |
| AFSM & MWA | Inverse variance weighted | -0.40962 | 0.124419 | 0.000994 | 0.663903 | 0.520232 | 0.847252 |
| AFSM & MWA | Weighted median | -0.26901 | 0.170308 | 0.114206 | 0.764133 | 0.547267 | 1.066936 |
| AFSM & MWA | MR Egger | 0.427411 | 0.534194 | 0.427676 | 1.533283 | 0.538154 | 4.368555 |
| AFSF & MWA | Inverse variance weighted | -0.32557 | 0.124319 | 0.008823 | 0.722114 | 0.565957 | 0.921357 |
| AFSF & MWA | Weighted median | -0.51177 | 0.182865 | 0.005132 | 0.599435 | 0.418875 | 0.857827 |
| AFSF & MWA | MR Egger | -0.2862 | 0.593067 | 0.631389 | 0.751116 | 0.234897 | 2.401793 |
| AFS & MOA | Inverse variance weighted | -0.40939 | 0.131826 | 0.001899 | 0.664053 | 0.51285 | 0.859836 |
| AFS & MOA | Weighted median | -0.51146 | 0.170001 | 0.002625 | 0.599622 | 0.429705 | 0.836729 |
| AFS & MOA | MR Egger | -0.68108 | 0.57079 | 0.234788 | 0.506071 | 0.165328 | 1.549096 |
| AFSM & MOA | Inverse variance weighted | -0.33724 | 0.132372 | 0.010845 | 0.71374 | 0.550634 | 0.92516 |
| AFSM & MOA | Weighted median | -0.28987 | 0.184551 | 0.116261 | 0.748363 | 0.521218 | 1.074497 |
| AFSM & MOA | MR Egger | -0.37594 | 0.583971 | 0.522854 | 0.686642 | 0.218597 | 2.156831 |
| AFSF & MOA | Inverse variance weighted | -0.42061 | 0.144787 | 0.003672 | 0.656646 | 0.494409 | 0.87212 |
| AFSF & MOA | Weighted median | -0.50568 | 0.193948 | 0.009126 | 0.603096 | 0.412377 | 0.88202 |
| AFSF & MOA | MR Egger | 0.691373 | 0.67878 | 0.313043 | 1.996454 | 0.527801 | 7.551763 |

AFS, age at first sexual intercourse; AFSM, age at first sexual intercourse of male; AFSF, age at first sexual intercourse of female; MWA, migraine with aura; MOA, migraine without aura; OR, odds ratio; LCI95, lower 95% confidence interval; UCI95, upper 95% confidence interval.

Supplement Table 5: IVW results of multivariate Mendelian randomization.

| **Exposure & Outcome** | **Beta** | **SE** | ***P* value** | **OR** | **OR_LCI95** | **OR_UCI95** |
| --- | --- | --- | --- | --- | --- | --- |
| AFS & Migraine | -0.32963 | 0.107517 | 0.002171 | 0.719192 | 0.582539 | 0.887902 |
| AFSM & Migraine | -0.29176 | 0.114073 | 0.010538 | 0.746946 | 0.597294 | 0.934094 |
| AFSF & Migraine | -0.24706 | 0.099112 | 0.012677 | 0.781096 | 0.64319 | 0.948572 |
| AFS & MWA | -0.35809 | 0.121763 | 0.003273 | 0.699009 | 0.5506 | 0.887421 |
| AFSM & MWA | -0.3648 | 0.149722 | 0.01483 | 0.694335 | 0.517753 | 0.931141 |
| AFSF & MWA | -0.26521 | 0.146795 | 0.070811 | 0.767042 | 0.575261 | 1.022761 |
| AFS & MOA | -0.36627 | 0.139631 | 0.008713 | 0.693315 | 0.52732 | 0.911563 |
| AFS & MOA | -0.19154 | 0.164064 | 0.243032 | 0.825691 | 0.598636 | 1.138863 |
| AFS & MOA | -0.3933 | 0.159483 | 0.013661 | 0.674829 | 0.493672 | 0.922462 |

AFS, age at first sexual intercourse; AFSM, age at first sexual intercourse of male; AFSF, age at first sexual intercourse of female; MWA, migraine with aura; MOA, migraine without aura; IVW, inverse variance weighted; OR, odds ratio; LCI95, lower 95% confidence interval; UCI95, upper 95% confidence interval.

Supplement Figure 1: Results (*P* value) of Horizontal pleiotropy and Heterogeneity


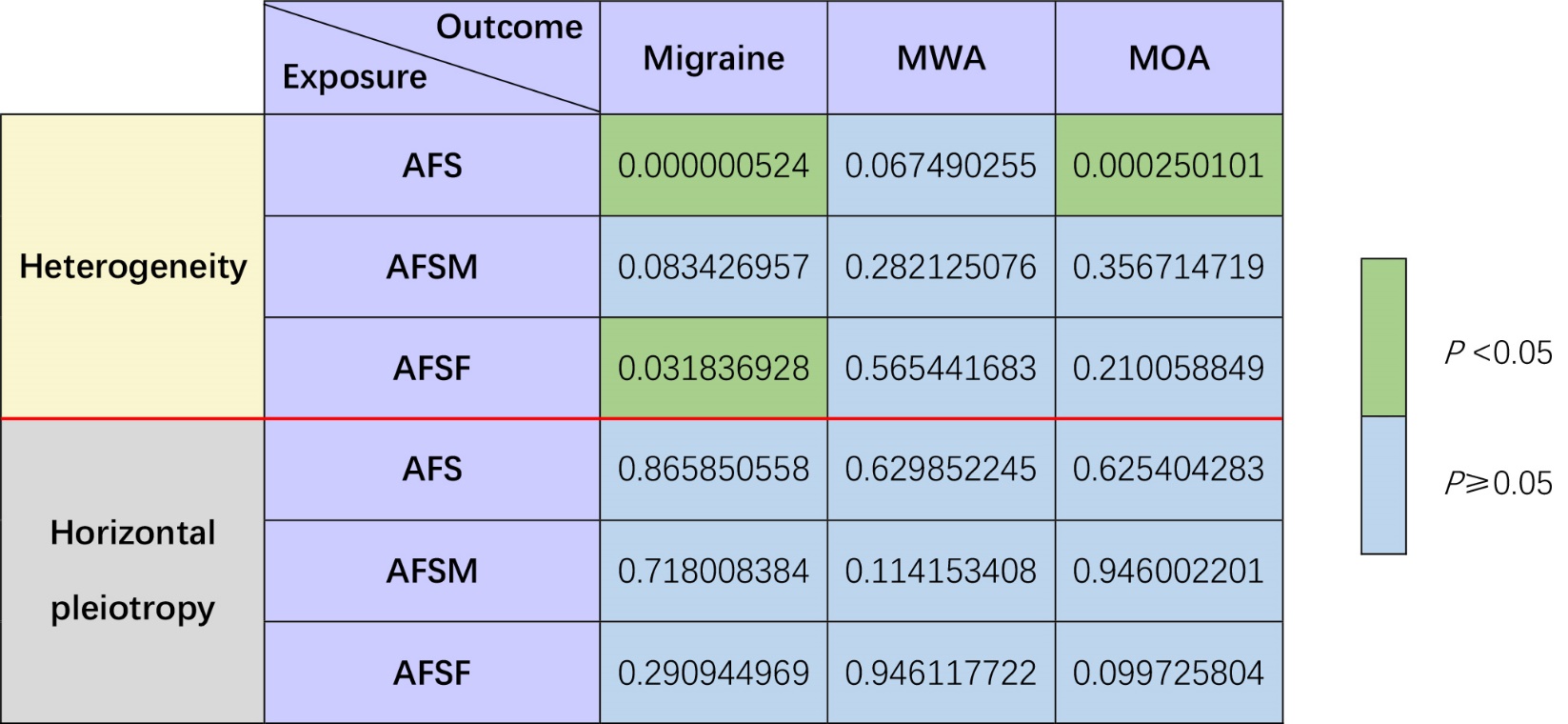


AFS, age at first sexual intercourse; AFSM, age at first sexual intercourse of male; AFSF, age at first sexual intercourse of female; MWA, migraine with aura; MOA, migraine without aura.

Supplement Figure 2: Results of leave-one-out analysis

Exposure & Outcome


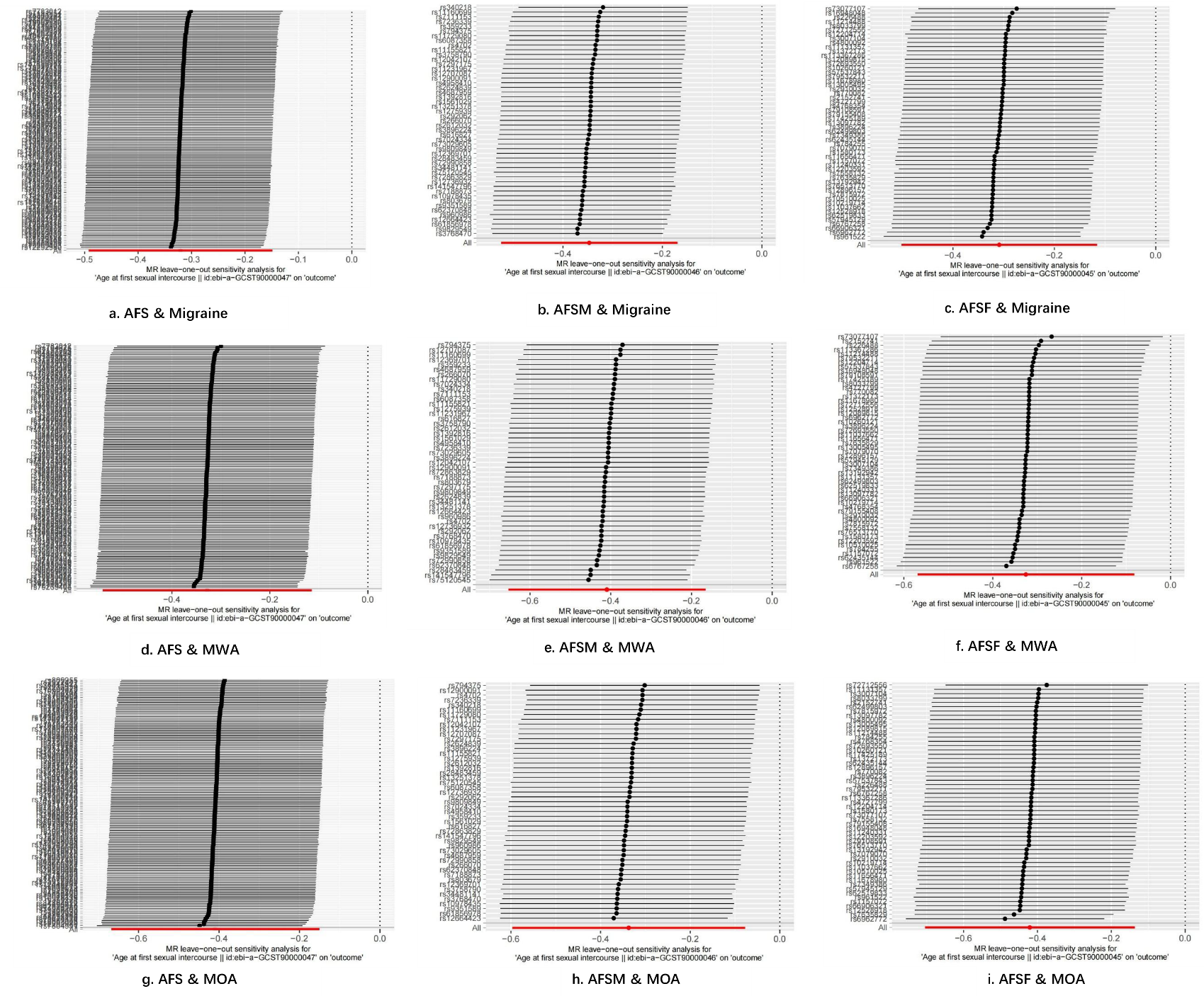


AFS, age at first sexual intercourse; AFSM, age at first sexual intercourse of male; AFSF, age at first sexual intercourse of female; MWA, migraine with aura; MOA, migraine without aura.

Supplement Figure 3: Results of funnel plot

Exposure & Outcome


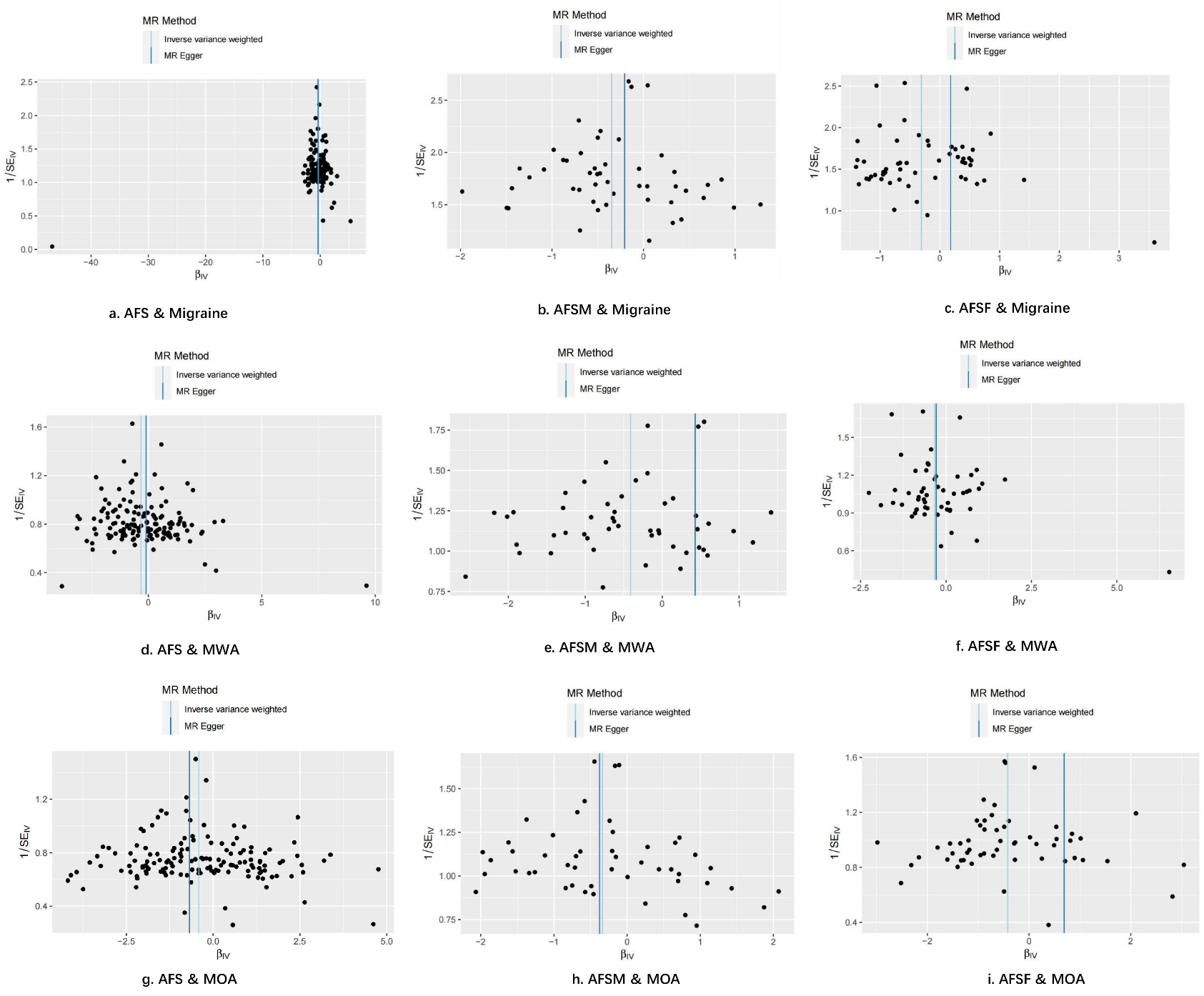


AFS, age at first sexual intercourse; AFSM, age at first sexual intercourse of male; AFSF, age at first sexual intercourse of female; MWA, migraine with aura; MOA, migraine without aura.

Supplement Figure 4: *P* values obtained from IVW for forward and reverse Mendelian randomization


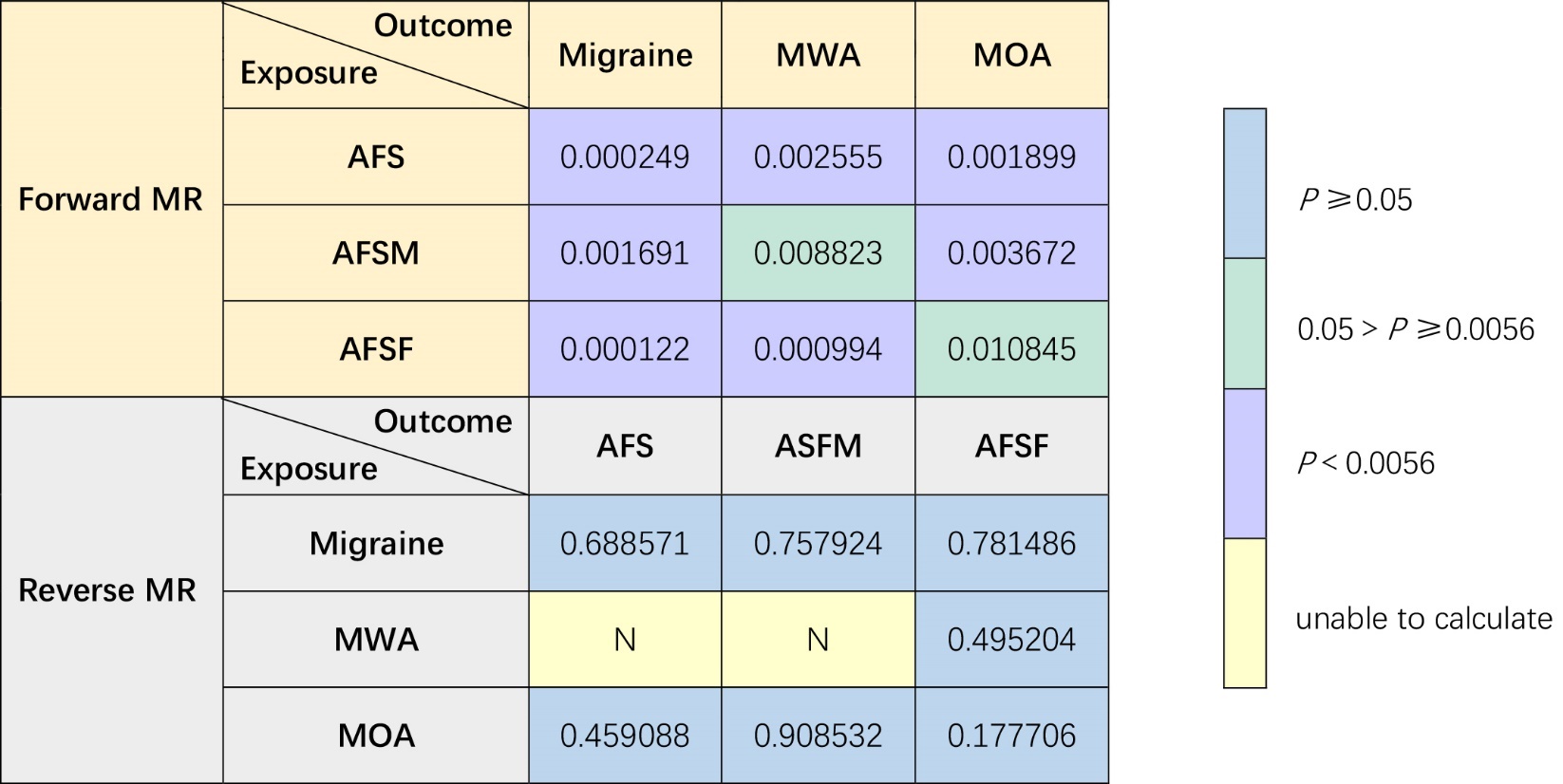


AFS, age at first sexual intercourse; AFSM, age at first sexual intercourse of male; AFSF, age at first sexual intercourse of female; MWA, migraine with aura; MOA, migraine without aura; MR, Mendelian randomization; IVW, Inverse variance weighted; N, P-value unable to calculate.
